# Harnessing Genomic Information for Identifying the Geographic Origin of Five North American Tree Species in Trade

**DOI:** 10.1111/eva.70295

**Published:** 2026-07-30

**Authors:** Pauline Hessenauer, Melanie Zacharias, Julien Prunier, Anna Fijarczyk, Roos Goessen, Julie Godbout, Anthony Piot, Isabelle Duchesne, Sam Yeaman, Ilga Porth, Nathalie Isabel

**Affiliations:** ^1^ Département des Sciences du bois et de la Forêt, Faculté de Foresterie, Géographie et Géomatique Université Laval, Pavillon Abitibi‐Price Québec Québec Canada; ^2^ Institut de Biologie Intégrative et des Systèmes, Université Laval, Pavillon Charles‐Eugène‐Marchand Québec Québec Canada; ^3^ Centre d'Étude de la Forêt, Université Laval Québec Québec Canada; ^4^ Institut intelligence et données, Université Laval, Pavillon Adrien‐Pouliot Québec Québec Canada; ^5^ Genovalia, Centre de valorisation des données en génomique non‐humaine, Université Laval Québec Québec Canada; ^6^ Université du Québec en Outaouais Ripon Québec Canada; ^7^ Institut des Sciences de la Forêt tempérée Ripon Québec Canada; ^8^ Centre de foresterie des Laurentides, Service Canadian des Forêts, Ressources naturelles Canada Québec Québec Canada; ^9^ Direction de la recherche forestière, Ministère des Ressources Naturelles et des Forêts Québec Québec Canada; ^10^ Faculté de Médecine Université Laval, Pavillon Ferdinand‐Vandry Québec Québec Canada; ^11^ Department of Biological Sciences University of Calgary Calgary Alberta Canada

**Keywords:** genomic assignment, machine learning, Single Nucleotide Polymorphism (SNP), traceability, wood products

## Abstract

Genomic tools for traceability of wood products offer a powerful tool to support sustainable forestry, fight illegal logging, and improve conservation efforts. Traditional identification methods (e.g., wood anatomy, spectroscopy) are limited in resolution or scope, but genomic approaches can infer both species identity and geographic origin. Using lodgepole pine ( *Pinus contorta* ) as a case study, we first compared three types of SNP datasets—random, adaptation‐linked, and machine learning‐selected—for their effectiveness to assign origin. While group‐based assignment methods (e.g., geographic or genetic) performed well with a small number of groups, their accuracy declined sharply as the number of groups increased, though it remained above random expectations even with 281 groups. To address this limitation, we developed a coordinate‐based methodological framework that directly predicts geographic origin from SNP marker sets of varying sizes. We applied this framework to four additional North American tree species ( *Populus trichocarpa* , black cottonwood; *P. tremuloides* , quaking aspen; *Picea mariana* , black spruce; *Pinus strobus* , eastern white pine), which represent a range of evolutionary histories and population structures. Using both classical and machine learning algorithms, including Linear Model (LM), K‐Nearest Neighbor (KNN), Random Forest (RF), and Gradient Boosting (GB), we predicted latitude and longitude with mean distance errors ranging from 15 to 383 km, depending on the species. Accuracy varied depending on species‐specific attributes such as population structure and sampling density. KNN performed best in datasets with high sampling density, such as *P. trichocarpa* , while GB achieved superior performance in more genetically homogenous taxa like *P. strobus* . This flexible, data‐driven approach enables precise traceability across tree species and supports forensic, regulatory, and certification uses. We outline practical guidelines emphasizing accurate taxonomic identification, broad sampling, and the use of ~2000–5000 random genomic markers. Although KNN and Gradient Boosting generally performed well, species‐specific differences warrant testing multiple algorithms.

## 1 Introduction

Effective wood and wood products tracking is crucial for promoting forest sustainability and combating deforestation, which helps to protect carbon‐storing forests, reduce greenhouse gas emissions, and mitigate the impacts of climate change. According to Interpol (International Policing Organisation), the transnational trade in illegally logged timber has an estimated value between 51 and 152 billion USD annually (Zacharias 2024; Interpol. Forestry Crime 2025). Canada imported about $6.7 billion and exported about $27.6 billion worth of wood products in 2023, making it one of the world's largest wood product exporters (Source: Forest Statistical Canada, consulted May 16, 2025). A study by Wiedenhoeft et al. (2019) employed forensic wood anatomy to examine 73 consumer wood products from major U.S. retailers and found that 62% contained at least one instance of mismatch, either intentional (fraudulent) or unintentional (misrepresented) claims regarding species identity and product type. These findings reveal a troubling gap between the widespread occurrence of such fraud and the limited capacity of current forensic wood identification systems to detect it. In response, several international laws and regulations have been established to combat illegal logging and curb the trade in illegally harvested timber, such as the Convention on International Trade in Endangered Species of Wild Fauna and Flora (CITES) which is signed by 185 parties and regulates transnational trade of protected and endangered species. However, documented cases of illegal logging demonstrated that species not listed under CITES can still be harvested illegally, often through fraudulent permits or logging outside authorized periods (Harvey 2020, The Guardian). In fact, any tree species can be subject to illegal exploitation, including through poaching in public or private forests (e.g., Dormontt et al. 2020; Cronn et al. 2021; Bourgon 2022, Canadian Geographic). This highlights the limitations of international regulations, which only cover a small fraction of commercially traded species.

Given the limited scope of international regulations, additional mechanisms are required to ensure legality and sustainability throughout the forest supply chain. Forest certification, a key voluntary mechanism based on third‐party verification, promotes sustainable forest management globally (Fraser 2007; Wyatt and Bourgoin 2010). It provides a framework for upstream compliance, reducing the need for intensive downstream enforcement, and contributes to greater public acceptance of forest resource use (Wilson et al. 2001). However, reliable certification depends on the robust traceability of wood products throughout the supply chain (Fernholz et al. 2021).

Traditional wood product certifications, typically managed through paper permits, labels, and audits, are vulnerable to tampering and mislabelling (Migone and Howlett 2012). These vulnerabilities have driven growing interest in technology‐based traceability systems capable of providing independent and verifiable evidence of origin. In contrast, advanced traceability systems relying on chemical or genomic signatures already implemented in other sectors to validate origin (e.g., wines), quality (e.g., food products), and certification status (e.g., coffee, cacao) (for a review see Godbout et al. 2018) remain underdeveloped but highly relevant in forestry. These systems are essential for screening wood products, protecting buyers, and enabling prosecution of illegal logging (Dormontt et al. 2015). Furthermore, they allow reputable timber traders to monitor their supply chains, ensuring compliance with origin and legality requirements before export. More recently, blockchain technology has gained attention as a tool in combating illegal logging. This secure digital ledger records and links each step in a product's journey, from forest to consumer, aiming to ensure transparency and prevent fraud (He and Turner 2022). While promising, blockchain faces practical challenges, such as data input reliability (Cheong 2025), and need to be effectively supplemented by independent verification tools to validate claimed timber origins (Komdeur and Ingenbleek 2021).

Implementing effective traceability systems requires reliable methods to identify the tree species from which wood products are derived and, in some cases, their geographic origin. Identification methods can be broadly grouped into three categories based on the type of information they generate. Visual identification, which relies on anatomical features of wood tissues and cells, is often limited to genus‐level resolution (Dormontt et al. 2015; Godbout et al. 2018). Chemical methods, encompass mass spectrometry analyses of wood extract composition (e.g., wood metabolites, lignin profiles), which are most commonly used for species identification, and stable isotope analyses, which rely on spatial variation in isotope ratios and are primarily used to determine the geographic origin (Dormontt et al. 2015; Flaig et al. 2024; Mortier et al. 2024; Price et al. 2025). In contrast, genetic methods, such as DNA barcoding and population‐based genomic approaches, offer precise taxonomic identification and can assign individuals to their geographic origin at the level of regions or biogeographic units, and in some cases even at their parental source populations (Ogden and Linacre 2015; Godbout et al. 2017, 2018, see figure 2). However, all these approaches rely on the availability of robust and comprehensive reference databases to produce accurate and reliable results.

DNA barcoding has been successfully used to assign samples to the genus or species levels in animals (e.g., birds [Kerr et al. 2007] or fishes [Ward et al. 2005]). Its application to plants and trees has emerged more recently, particularly for high‐value or endangered species like mahogany ( *Swietenia macrophylla* ) or rosewoods (*Dalbergia* species) (Höltken et al. 2012; Yu et al. 2017). Beyond species identification, the use for DNA‐based markers to infer geographic origin is rapidly expanding. Specifically, the development of Single Nucleotide Polymorphism (SNP) arrays has enabled geographic assignment in tree species such as demonstrated in Spanish cedar ( *Cedrela odorata* ) and bigleaf maple ( *Acer macrophyllum* ) (Finch et al. 2020; Cronn et al. 2021). In these studies, approximately 150 SNPs were sufficient to predict ecoregions or countries of provenance with good accuracy. However, assignment to finer‐scale localities remained challenging, with substantially higher error rates, underscoring the difficulty of high‐resolution geographic inference using relatively small marker sets (Finch et al. 2020; Cronn et al. 2021).

Beyond law enforcement and timber trade monitoring, accurate geographic origin identification is also critical in forest management contexts where provenance influences adaptation, performance, and long‐term sustainability. In regions where seed collection and the use of forest reproductive material are governed by strict provenance policies, the ability to determine and/or certify the origin of seedlings prior to deployment is therefore essential. In Canada, such policies are implemented in several provinces to ensure local adaptation and sustain forest productivity, as illustrated by recent case studies in white spruce and lodgepole pine (Godbout et al. 2017; Peery et al. 2022). In addition, the need for reliable long‐term tracking of deployed seedlings is expected to increase under assisted migration strategies, further strengthening the case for robust genomic approaches for origin identification and monitoring (Wotherspoon et al. 2025).

The diversity in methodologies and genetic markers used to develop SNP panels, often tailored to specific species' evolutionary contexts, raises a crucial question: can a single assignment method or algorithm effectively accommodate the genetic complexity across multiple species? Addressing this, a critical need exists for a standardized pipeline to identify candidate SNPs suitable for panel design across both deciduous and coniferous tree species. Such a standardized approach would be particularly valuable for genomic assignment in detecting illegal trading of protected tropical species (Ng et al. 2022; Meyer‐Sand 2024). Tropical species, often characterized by more fragmented and less continuous distributions than temperate species, typically exhibit high population divergence (Eo et al. 2008), making them prime candidates for precise, marker‐based geographic origin assignment.

From both policy and statistical perspectives, a crucial distinction exists between verifying and determining the origin of a species, with the latter demanding a significantly more elaborate approach (Godbout et al. 2018; Deklerck 2023). Verification assesses whether a sample matches a known reference or predefined group, often relying on a limited number of key genetic markers sufficient for classification. In contrast, determining geographic origin requires a more extensive dataset. This is because origin determination is an inference problem, aiming to deduce spatial or evolutionary patterns rather than simply assigning samples to predefined categories. This complexity arises from the need to account for population structure, gene flow, and local adaptation, all of which necessitate high‐resolution genetic information and analytical approaches capable of handling high‐dimensional genetic data (Godbout et al. 2018).

Machine learning (ML), a subcomponent of Artificial Intelligence, is increasingly vital in population genomics for deciphering complex biological patterns and enhancing predictive accuracy (Libbrecht and Noble 2015). ML facilitates tasks such as classifying individuals into taxonomic groups based on genetic markers (Yu et al. 2017; He et al. 2019) and predicting geographic origins by analyzing spatially structured genetic variation (Battey et al. 2020). These models are trained on labeled datasets to discern underlying relationships within the data (Whalen et al. 2022). The selection of an appropriate ML algorithm is important, as different algorithms possess unique assumptions and performance characteristics that significantly influence the accuracy and reliability of the prediction outcomes (Libbrecht and Noble 2015).

In this study, we utilized well‐characterized genomic data from forest tree species of known origin and identity to develop and validate an individual‐level tree assignment pipeline. Our objectives were to: (i) develop a genomics‐informed traceability system leveraging ML approaches; (ii) establish general guidelines for tree genomic assignment strategies, considering factors like SNP number/type and interspecific hybridization impact; and (iii) identify the most reliable methods for assigning individual trees to their geographic origin with high precision across diverse species and scales. We initiated our investigation with a proof‐of‐concept case study in lodgepole pine, assigning individuals to discrete groups. Subsequently, to assess broader applicability in predicting geographic origin, we extended this approach to five native Canadian tree species—three coniferous ( *Picea mariana* (Mill.) B.S.P., *Pinus contorta* (Douglas ex Loudon), *Pinus strobus* (L.)) and two broadleaf trees ( *Populus trichocarpa* (Torr. & A. Gray ex Hook.) and *Populus tremuloides* (Michx.)). These species exhibit post‐glacial geographical clustering and varying degrees of population genetic structure driven by isolation‐by‐distance and/or local climatic adaptation (Godbout et al. 2008; Prunier et al. 2011, 2013; Geraldes et al. 2014; Nadeau et al. 2015; Porth et al. 2015; Goessen et al. 2022). Given their genetic characteristics and the extensive genomic resources (particularly large SNP datasets), we hypothesized that our developed ML‐based pipeline could accurately predict the geographic location of samples of unknown origin.

## 2 Materials and Methods

### 2.1 Data Acquisition

**FIGURE 1:**
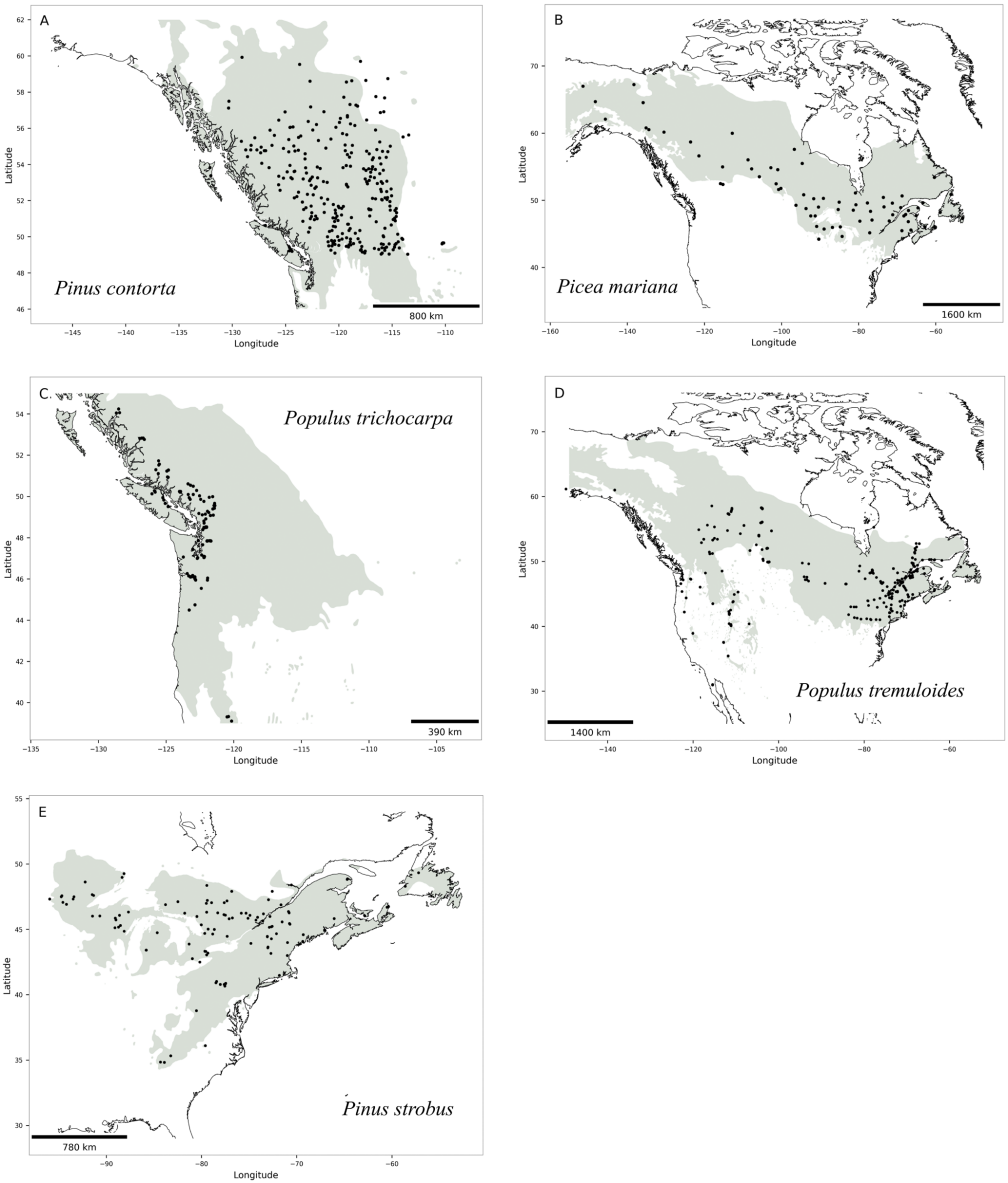
Sampling distribution of five tree species across North America. Each dot represents a sampling location for (A) *Pinus contorta* , (B) *Picea mariana* , (C) *Populus trichocarpa* , (D) *Populus tremuloides* , (E) *Pinus strobus* . Shaded areas represent the species' geographic distribution range.

In this study, we focused on five commercially important tree species in Canada ( *Pinus contorta* , *Picea mariana* , *Populus trichocarpa* , *Populus tremuloides* , and *Pinus strobus* ), each valued for distinct uses and primarily harvested from public lands. These species were selected not only for their economic relevance, but also because genomic resources based on SNPs were either developed or under development (see below). Genotyped individuals were georeferenced, including hundreds of individuals spanning most of the species' natural distribution range (Figure 1).

*Pinus contorta* (lodgepole pine) is native to western North America and comprises two main subspecies: the coastal *P. contorta* ssp. *contorta* and the continental *P. contorta* ssp. *latifolia* . Its genetic structure reflects several distinct lineages associated with multiple glacial refugia (Godbout et al. 2008). In central Alberta, *P. contorta* comes into contact with *Pinus banksiana* (jack pine), leading to hybridization between the species (Burns et al. 2019). This hybrid zone is characterized by elevated population differentiation and significant introgression (Godbout et al. 2012; Cullingham et al. 2013). Datasets (trees genotyped using SNP markers) were obtained from georeferenced samples collected in two provenance trials established in British Columbia and Alberta, respectively, and published in Mahony et al. (2019). In forestry, provenance trials are commonly used to assess the performance of trees from different geographic origins when grown in a shared environment. Briefly, the dataset comprised 1594 lodgepole pine seedlings from a raised‐bed common garden at Totem Field (UBC, Vancouver), representing 281 provenances (hereafter named sampling locations) spanning the climatic range of *P. contorta* in British Columbia and Alberta, including both *latifolia* and *contorta* subspecies and the hybrid zone with jack pine ( *P. banksiana* ). A complementary set of 1906 seedlings from the same sampling locations grown in a controlled growth‐chamber experiment brought the total to 3500 genotyped individuals (median 11 seedlings per provenance, range 7–24). All individuals were genotyped using the AdapTree 50 K Axiom SNP array. A total of 36,384 SNPs were retained after filtering, including 3934 intergenic control SNPs and 32,407 candidate adaptive SNPs.

*Picea mariana* (black spruce) is a North American conifer with an extensive distribution across the boreal forest, spanning from coast to coast (Chagnon et al. 2022). Despite this broad range, its population genetic structure is relatively simple, consisting of only three major genetic clusters shaped by historical glacial vicariance (Prunier et al. 2012; Fijarczyk et al. 2025). In parts of its range, black spruce occurs in sympatry with its close relative *Picea rubens* (red spruce), and introgressive hybridization between the two species has been documented (Perron and Bousquet 1997; de Lafontaine et al. 2015). Genomic data used in this study were obtained from Fijarczyk et al. (2025), who genotyped over 2000 individuals from 70 sampling locations. Plant materials were collected from four provenance trials established by Natural Resources Canada as part of the Range‐wide Provenance Study (Morgenstern 1978; see Supplementary Methods for details). Putative hybrid individuals, with a *Q*‐value ranging between 0.57 and 0.94, were discarded to ensure that only genetically pure black spruce individuals were represented in our analyses.

*Populus trichocarpa* (black cottonwood) occupies riparian habitats throughout western North America from Baja California to Alaska. It exhibits high levels of genetic diversity (Evans et al. 2014; Piot et al. 2019) and displays a continuous pattern of genetic differentiation across its range, even at fine spatial scales (Slavov et al. 2010, 2012). This global population structure is the result of neutral processes such as isolation‐by‐distance and a strong genetic drift caused by small effective population sizes (Porth et al. 2015). In the northeastern part of its range, *P. trichocarpa* hybridizes with *Populus balsamifera* , leading to introgression along the species' distribution margins. All hybrids with other *Populus* species were removed from this dataset based on visual inspection of a principal component analysis, thereby only representing *P. trichocarpa* (Suarez‐Gonzalez, Hefer, Lexer, Cronk, and Douglas 2018; Suarez‐Gonzalez, Hefer, Lexer, Douglas, and Cronk 2018; Piot et al. 2019). The published dataset (trees genotyped using SNP markers) was obtained from Piot et al. (2019), and covers 95 sampling locations across the range of the species.

*Populus tremuloides* (quaking aspen) is a widely distributed broad‐leaf tree, ranging from Canada to central Mexico, and thrives across a broad spectrum of environmental conditions. The species exhibits high levels of intraspecific genetic diversity (Wang et al. 2016) and is known to hybridize with *P. grandidentata* in the northeastern part of its range (Deacon et al. 2019). A recent population genomic study has identified four major genetic lineages across its range, each showing adaptive differentiation to temperature and precipitation gradients (Goessen et al. 2022). We utilized this well‐characterized genomic dataset published in Goessen et al. (2022), which includes SNP‐genotype trees from various sampling locations across the range of the species. This dataset was already filtered to exclude polyploid individuals and admixed individuals (for more details see Goessen et al. 2022).

*Pinus strobus* (eastern white pine) is an eastern North American conifer that exhibits important genetic diversity despite extensive historical harvesting and widespread outbreaks of white pine blister rust (Mehes et al. 2009). *P. strobus* exhibits weak spatial genetic structure across its range; indeed, its natural distribution range is smaller than that of *P. mariana* or *P. tremuloides* (both transcontinental), and it spans an area with fewer major physiographic barriers compared to the species from the Pacific Northwest (such as *P. contorta* and *P. trichocarpa* ) (Marquardt et al. 2007; Nadeau et al. 2015). Plant materials for genomic analysis were collected from various sources (provenance‐progeny trials, seed orchard, and juvenile seedlings [see Nadeau et al. 2015 and Supplementary Methods for details]).

For all five species, we had access to genomic data, including SNPs from both coding and noncoding regions. These datasets comprised both previously published and newly generated data. For *P. contorta* , we used data from (Mahony et al. 2019), comprising 32,407 SNPs genotyped in 3500 individuals across 281 sampling locations (Figure 1A). For *P. mariana* , we used existing genotype data (Fijarczyk et al. 2025); details for these datasets are provided in the Supporting Information. This resulted in 29,978 SNPs for 2482 individuals for the *P. mariana* dataset (Figure 1B). The original *P. trichocarpa* dataset consisted of whole‐genome resequencing based genotype data from over 1000 individual accessions published by (Piot et al. 2019, 2024), but was reduced to 29,840 SNPs and 601 individual genotypes. From the total of 1000 genomes available in Piot et al. (2019), 601 individuals were first selected according to the availability of the geographic coordinates of origin (Figure 1C). Then, SNPs were selected according to calling quality described in the vcf file and tolerating a maximum of 20% of missing data, and minor‐allele‐frequency as low as 1%. Finally, the 28,840 SNPs were selected in order to be regularly distributed along the entire genome. Similarly, for *P. tremuloides* , we used genotype data for 600 individuals and selected 12,434 SNPs out of the 37,519 available (Figure 1D). For *P. strobus* , we used newly generated genotype data that had not been previously published or analyzed; details for these datasets are provided in the Supporting Information. This resulted in 9278 SNPs for 617 individuals for the *P. strobus* dataset (Figure 1E). All datasets were analysed separately.

### 2.2 Classification‐Based Assignment to Genetic and Geographic Groups in Lodgepole Pine

To assess the feasibility and limitations of SNP‐based geographic assignment for each studied tree species, we first conducted a series of preliminary tests prior to finalizing the bioinformatic pipeline. We aimed to test the effect of marker type used (i.e., genotype‐environment association (GEA) outliers, randomly selected and pre‐selected by AI) on assignment accuracy to predefined genetic or geographic clusters. To do so, we used the lodgepole pine dataset, as it provides a good representation of the species' genetic diversity across the majority of its distribution range in Canada (Mahony et al. 2019). Furthermore, this dataset was extended to describe the genotype‐environment associations, thus providing an opportunity to explore these associations in group assignment (Yu et al. 2022). Missing data was imputed using *gl.impute* function from dartR (Mijangos et al. 2022) in R (R Core Team, n.d.), based on allelic frequencies.

We assigned individuals to genetic clusters using STRUCTURE (Pritchard et al. 2000), starting with *K* = 3 (Figure 1), which corresponds to the three main genetic clusters previously described in *P. contorta* (Godbout et al. 2008). Higher values were then tested (*K* = 4, not shown; *K* = 5, Figure S1) to evaluate whether increasing the number of genetic clusters improved assignment accuracy. We used the Random Forest (RF) classification algorithm implemented in the *randomforest* package (Liaw and Wiener 2002) in R (R Core Team, n.d.) after dividing the data into 80% and 20% for training and validation sets, respectively. We emphasize that although STRUCTURE and Random Forest analyses are based on the same SNP dataset, they address distinct analytical objectives: STRUCTURE infers population structure in an unsupervised framework, whereas RF evaluates how accurately this inferred structure can be recovered through supervised classification. Consequently, perfect assignment accuracy in RF models to a STRUCTURE‐defined genetic group is neither expected or biologically meaningful, as STRUCTURE does not define discrete, error‐free genetic groups. Indeed, individuals are rarely assigned with 100% probability to a single cluster due to ongoing gene flow, isolation by distance, and natural hybridization. Finally, because individual assignment is often performed using arbitrary geographic delineations (e.g., country, province, or region), we sought to mimic this common practice by also defining four geographic regions. To define balanced geographic clusters, we used the *k‐means* function implemented in R package *stats* (R Core Team, n.d.) with 4 centers on the sampling location's geographic coordinates (Figure S2).

For each clustering tested (genetic: *K* = 3, *K* = 4, *K* = 5, geographic: *K* = 4, and 281 sampling locations), we first built the model utilizing the complete SNP dataset. This approach allowed us to estimate accuracy metrics as well as generate a comprehensive list of SNP markers ranked by their relative importance. A higher importance means that a given SNP has a larger effect in the model. To push assignment performance to its limits, we extracted the top 5 and 10 most important SNPs and used them as pre‐selected “AI” markers to evaluate model performance with an extremely small number of markers. Following this, we retrieved the GEA candidate SNPs to build the climate outliers dataset (Yu et al. 2022). Finally, we randomly sampled a total of 5000 SNPs from the full dataset to generate the random dataset.

As indicated above, lodgepole pine naturally hybridizes with jack pine. Thus, to assess the impact of SNP selection and genetic clustering on assignment accuracy, individuals identified as genetically admixed (membership coefficient < 0.8) were excluded based on Structure results for *K* = 2, *K* = 3, and *K* = 4. For each value of *K*, RF classification models were trained on the remaining “pure” individuals using subsets of the top‐ranked SNPs, ordered by importance in an initial model. SNPs subsets ranged from the top 10 to the top 1000. For each subset, data were partitioned into 80% training and 20% testing sets, and models were fitted using 10,000 trees. Assignment accuracy was calculated as the proportion of individuals correctly classified in the test set.

Model accuracy was assessed by a confusion matrix, which summarizes the number of correct and incorrect classifications for each group, allowing the calculation of the proportion of correctly classified individuals across all groups (= overall classification accuracy). To allow meaningful comparisons across models classifying into different numbers of sampling locations (i.e., classification tasks of varying complexity), we also computed the Improvement over Chance Index (ICI) (Equation 1). This index, similar to Cohen's kappa (Cohen 1960), quantifies the proportion of possible improvement over random classification achieved by the model, accounting for the baseline accuracy expected under random assignment (1 divided by the number of groups). An ICI value close to 1 indicates strong model performance relative to chance, whereas values near 0 suggest performance similar to random guessing.

(1) $$\mathrm{ICI}=\frac{\text{Model accuracy}-\text{Random chance accuracy}}{1-\text{Random chance accuracy}}$$

### 2.3 Coordinate‐Based Geographic Assignment Across Five North American Tree Species

**FIGURE 2:**
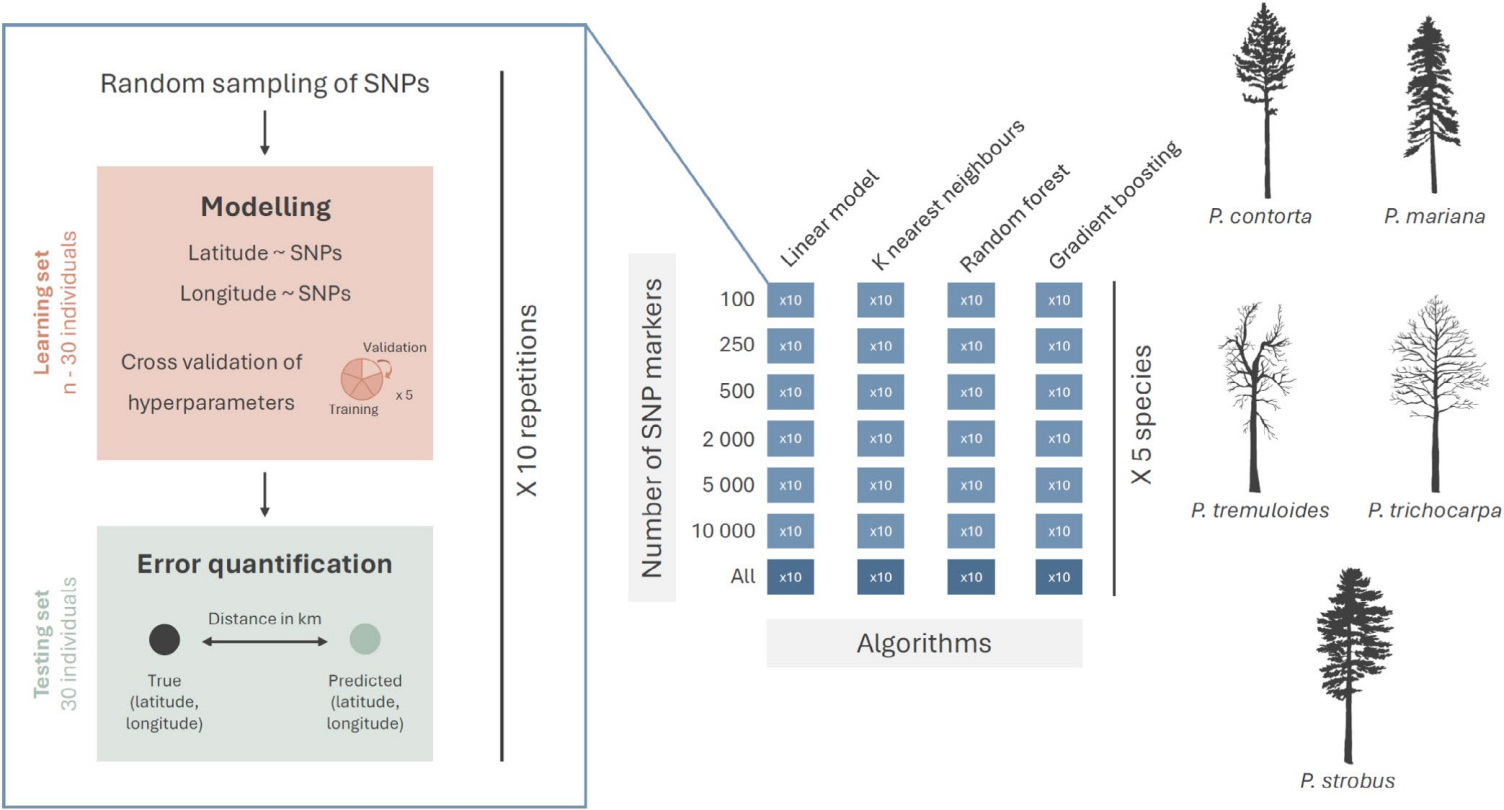
Analytical workflow: Coordinate‐based genomic assignment pipeline. Random subsets of SNPs of increasing size were used to train models predicting geographic coordinates (latitude and longitude). For each iteration, samples were split into a learning set (n − 30 individuals) and a testing set (30 individuals), and model hyperparameters were optimized using cross‐validation. Prediction performance was quantified as the geographic distance (km) between the true and predicted coordinates. This workflow was repeated 10 times across multiple marker densities and evaluated using four algorithms (LM, KNN, RF, GB), independently for each tree species.

We developed a ML assignment pipeline to evaluate the predictive accuracy of different optimized models for independently estimating latitude and longitude of a given sample, using random subsets of SNPs of varying sizes (Figure 2). This pipeline was implemented in Python using *scikit‐learn* v.1.1.2 library (Pedregosa et al. 2011). To benchmark model performance, we compared several ML algorithms against a linear regression model (LM), used as a baseline. The evaluated algorithms included *k*‐nearest‐neighbors (KNN), the Random Forest (RF) regression, and Gradient Boosting (GB) regression. LM (Galton 1886) minimizes the sum of squared differences between observed and predicted values and provides a classical, interpretable statistical approach. The KNN algorithm predicts target values based on the average response of the *K* nearest samples in the feature space (Guo et al. 2003). RF (Ho 1995) constructs an ensemble of decision trees and generates predictions by averaging across trees, while GB (Friedman 2001) builds trees sequentially, with each new tree correcting errors made by the previous ones to iteratively reduce prediction error.

We included RF and GB because of their ability to capture complex, nonlinear relationships and interactions among predictors, albeit with an increased risk of overfitting. In contrast, LM provides a simpler, more interpretable model that may generalize better in some scenarios. KNN was incorporated as a nonparametric, instance‐based learning method that makes no assumptions about the underlying data distribution and can capture local spatial patterns potentially missed by other models.

Within each species dataset, we evaluated model performance using a randomly selected test set of 30 individuals (Figure 2). Hyperparameter optimization was then conducted on the remaining training set using a comprehensive grid search approach. For each hyperparameter combination (Table S1), the learning set was partitioned into five folds. Models were trained on four folds (80%) and validated on the remaining fold (20%). This procedure was repeated five times, each time with a different validation fold, and performance scores were averaged across folds. The hyperparameter combination yielding the best average performance was selected as the optimal configuration using the mean squared error (MSE) as the optimization metric.

For each iteration, defined by a combination of SNP set, SNP count, and algorithm, the model was trained, optimized, and the corresponding optimal hyperparameter values were recorded (Table S2). Final model accuracy was assessed as the geographic distance (in kilometers) between observed and predicted coordinates, calculated from geographic coordinates while accounting for the decreasing longitudinal distance per degree at higher latitudes. Analyses were conducted using randomly subsampled SNP of varying sizes (10, 100, 250, 500, 2000, 5000, 10,000, and the full dataset). For each SNP subset size, the entire modeling and evaluation procedure was repeated 10 times, each time using a different randomly drawn SNP subset from the complete dataset.

The most important SNPs contributing to geographic location prediction were identified using the permutation feature importance procedure. In this approach, feature values are reshuffled one feature at a time (while keeping all other features unchanged), and the resulting decrease in model performance is evaluated; a greater decline in performance indicates higher feature importance. This procedure was applied to the final best‐performing model for each species. Because permutation feature importance is model‐agnostic, it is compatible with all algorithms evaluated in this study.

To enable meaningful comparisons of prediction accuracy across datasets with differing spatial sampling densities, we computed a Relative Spatial Error (RSE) index. For each dataset, we first calculated the mean pairwise geographic distance between sampling locations using R package geodist (Padgham and Sumner 2025), which implements the Haversine formula. RSE was then calculated by scaling the mean geographic prediction error by the mean distance between sampling locations within the dataset (Equation 2). An RSE value less than 1 indicates that the model predicts coordinates with greater precision than the natural spacing of sampling locations, while values above 1 indicate lower spatial resolution in geographic assignment.

(2) $$\mathrm{RSE}=\frac{\text{Mean prediction error distance}}{\text{Mean distance between sampling locations}}$$

## 3 Results

### 3.1 Assignment Accuracy Depends on Marker Choice and Group Resolution

To test the use of genomic markers for assigning trees to their geographic origin we first investigated the effect of marker type and number, the type and number of groups considered, and the presence of hybridization in lodgepole pine (Table 1, Figure S3). Assignment accuracy and the ICI metric were calculated for predictions of individual membership to genetic groups (*K* = 2 or *K* = 4), geographic groups (*n* = 4), or their original sampling locations (*n* = 281). Overall, classification accuracy was highest when assigning individuals to the two genetic groups (*K* = 2), with accuracy consistently exceeding 80% across all SNP datasets and peaking at 94.07% when using the entire SNP dataset (32,000 SNPs), accompanied by high ICI values (0.84–0.88), well above the random chance accuracy of 50%. A similar trend was observed when classifying individuals into four genetic groups (*K* = 4), where the model achieved up to 87.28% accuracy and an ICI of 0.83 using the whole SNP dataset, and maintained good performance with the top 100 SNPs (86.21% accuracy, 0.82 ICI). For geographic group assignment (*n* = 4), accuracy values were slightly lower but remained above random expectation (25%), with the highest accuracy observed when using the top 100 SNPs (68.35% accuracy, 0.58 ICI) and the full SNP dataset (66.9% accuracy, 0.56 ICI). Fine‐scale assignment to the 281 original sampling locations proved to be more difficult, with accuracy not exceeding 6.8% even when using the full SNP set (compared to 0.36% random expectation), though still yielding informative patterns. It is noteworthy that even with as few as 100 top‐ranked SNPs, the model retained a substantial portion of the accuracy achievable with the full dataset, particularly when assigning individuals to genetic and geographic groups. For instance, assignment to four genetic groups with the top 100 SNPs reached 86.21% accuracy, closely matching the 87.28% achieved with 32,000 SNPs. This demonstrates the potential of a relatively small set of highly informative markers to effectively capture population structure and geographic patterns, offering a promising approach for streamlined assignment models in future applications.

**TABLE 1:** Assignment accuracy in lodgepole pine (*Pinus contorta*) as a function of cluster definitions and marker selection.

Dataset	Assigning to *K* = 2 genetic groups		Assigning to *K* = 4 genetic groups		Assigning to *n* = 4 geographic groups		Assigning to the 281 original sampling locations	
	Model accuracy	ICI	Model accuracy	ICI	Model accuracy	ICI	Model accuracy	ICI
860 GEA outliers	92.34	0.85	80.05	0.73	60.54	0.47	3.32	0.03
5000 random SNPs	92.77	0.86	78.03	0.71	66.18	0.55	4.91	0.05
Whole dataset (32,000 SNPs)	94.07	0.88	87.28	0.83	66.9	0.56	6.80	0.06
Top 100 SNPs	92.05	0.84	86.21	0.82	68.35	0.58	5.00	0.05
Top 10 SNPs	80.08	0.60	55.63	0.41	49.36	0.32	1.42	0.01
Top 5 SNPs	80.96	0.62	51.02	0.35	43.11	0.24	1.30	0.01
Accuracy under random chance	50		25		25		0.36	

*Note:* Model absolute accuracy percentage is represented in column model accuracy (percentage of correctly assigned individuals in the confusion matrix) and ICI column which represents how much better than a random prediction the model is. Random assignment success in the bottom line represents the percentage of accuracy that would be obtained by chance.

Given the reduced accuracy when assigning individuals to 281 predefined sampling locations and the need for fine‐scale geographic resolution, we chose to predict continuous geographic coordinates rather than discrete categories. This approach aims to achieve more precise and scalable spatial predictions while maintaining our focus on the impact of markers on prediction accuracy.

### 3.2 Determinants of Spatial Prediction Accuracy Across Forest Tree Species

**FIGURE 3:**
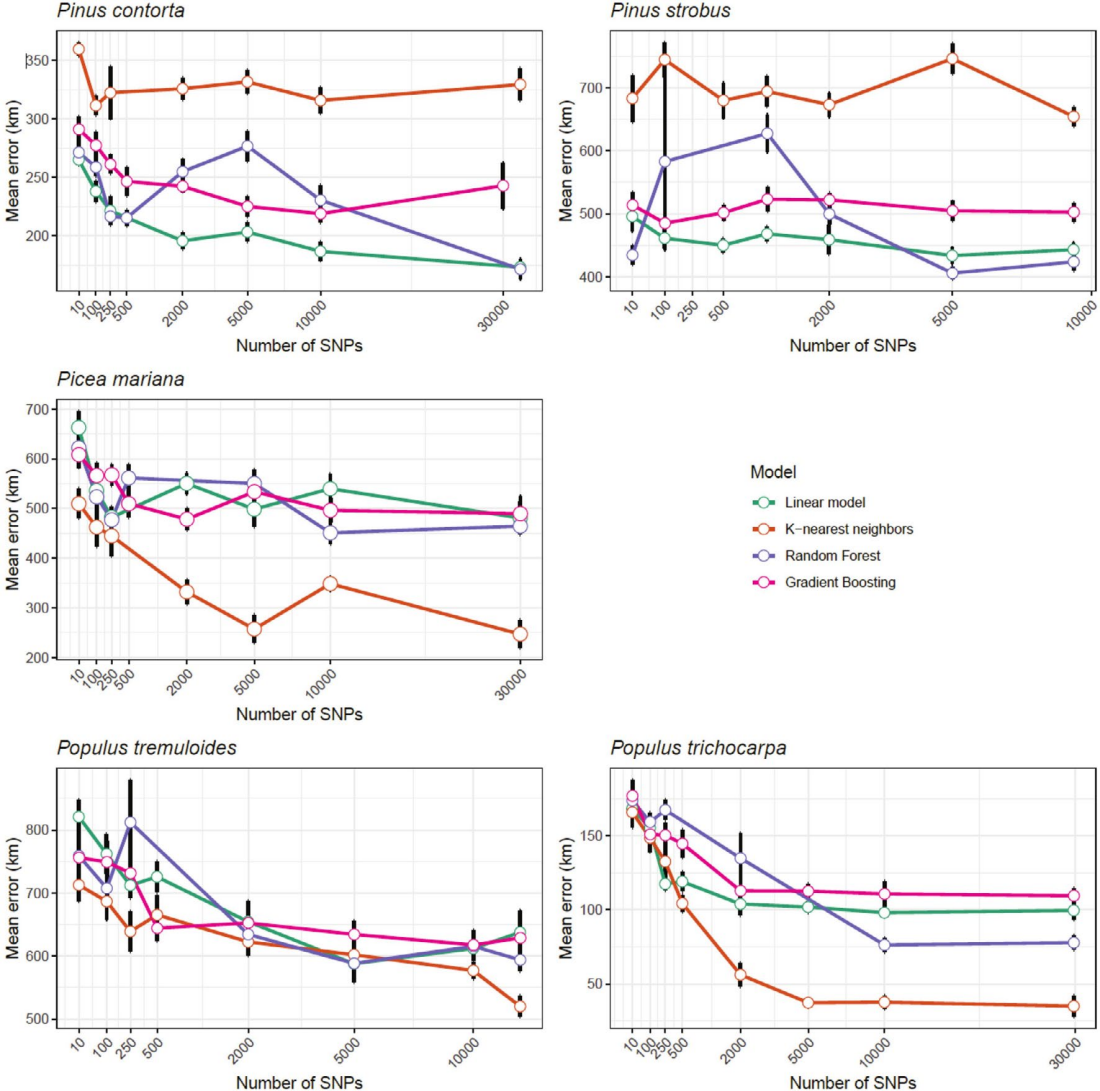
Mean geographic prediction error (km) as a function of the numbers of SNPs used to estimate individual coordinates. The *y*‐axis represents the mean geographic prediction error (km) across all individuals, averaged over ten repetitions, while the *x*‐axis indicates the number of subsampled SNPs used to train the model. Different algorithms are shown in different colors: LM (linear model; purple, baseline model), KNN (k‐nearest neighbors; orange), RF (random forest; pink), and GB (gradient boosting; green). Each point on the graph represents the mean error for a given number of SNPs and algorithm, with error bars indicating the standard deviation.

For all species, geographic prediction accuracy improved as the number of genetic markers included in the model increased (Figure 3). This gain was reflected by a consistent reduction in mean prediction error (km). However, a plateau effect was often observable, with some models reaching relatively stable error rates after an initial decline, suggesting diminishing improvements from adding additional SNPs. The best optimized algorithm differed across species. KNN achieved the lowest prediction error in three of the five species: *Picea mariana* , *Populus trichocarpa* , and *P. tremuloides* . In contrast, the two tree‐based machine learning algorithms, GB and RF, followed similar performance trends across all five species, with GB generally outperforming RF. This difference is likely due to GB's sequential tree‐building process, which optimizes performance more effectively than RF's random tree construction. The varying sizes of error bars indicate differences in prediction accuracy across replicates for a given number of markers. This variability was most pronounced when fewer genetic markers were included and tended to decrease as the number of SNPs was incorporated. This pattern suggests that increasing the number of markers enhances the likelihood of capturing informative SNPs, resulting in more consistent and reliable predictions.

The lowest geographic prediction error, measured as the minimum distance error (in km) for the best‐performing model, was observed in *P. trichocarpa* (15 km), followed by *P. mariana* (113 km), *Pinus contorta* (136 km), *P. strobus* (340 km), and *P. tremuloides* (383 km) (Table 2). However, our datasets differ significantly in the mean geographic distance between sampling locations (Table 2, Figure S4).

**TABLE 2:** Summary of datasets used for spatial prediction models across five tree species. For each species, we report the number of sampling locations (N loc), the number of individuals genotyped (N ind), the total number of SNP markers (N SNPs), and the mean number of individuals per sampling location, with minimum and maximum values (N ind/loc). Sampling density is described by the mean distance between sampling locations (Mean dist; km). *H*_o_, *H*_e_, *F*_ST_, and *F*_IS_ report overall statistics for observed heterozygosity, expected heterozygosity, genetic differentiation, and inbreeding coefficient, respectively. The minimum mean prediction error (in km) corresponds to the smallest average distance between predicted and true coordinates achieved by the best‐performing model. Relative spatial error (RSE) is calculated as the ratio of the minimum mean prediction error to the mean distance between sampling locations, providing a standardized metric for comparing spatial prediction accuracy across datasets.

	*Pinus contorta*	*Pinus strobus*	*Picea mariana*	*Populus tremuloides*	*Populus trichocarpa*
Common name	Lodgepole pine	Eastern white pine	Black spruce	Quaking aspen	Black cottonwood
Dataset source	Mahony et al. (2019)	See Supplementary Methods	Fijarczyk et al. (2025), see Supplementary Methods	Goessen et al. (2022), see Supplementary Methods	Piot et al. (2019)
Ploidy in the dataset	2*n*	2*n*	2*n*	2*n*	2*n*
*N* loc	281	110	70	102	95
*N* ind	3500	617	2482	460	601
*N* SNPs	32,000	9278	29,978	12,434	29,840
*N* ind/loc	12.5 (7–24)	4.3 (1–17)	35.4 (5–88)	5.9 (1–20)	6.2 (1–86)
Mean dist (in km)	496	867	1689	1880	260
*H*_o_	0.356	0.293	0.118	0.181	0.198
*H*_e_	0.25	0.295	0.21	0.19	0.191
*F*_ST_	0.01	0.034	0.062	0.11	0.051
*F*_IS_	−0.425	0.009	0.428	0.046	−0.039
Min mean error (in km)	136	340	113	383	15
RSE	0.27	0.39	0.07	0.20	0.06

Assignment accuracy varied among species, largely influenced by sampling density and sample size (Table 2, Figures S5–S9). RSE measured model prediction performance while including the effect of sampling density and ranged from 0.06 for *P. trichocarpa* to 0.39 for *P. strobus* , meaning that our models performed better than random guessing based on nearest sampling distances (RSE < 1). *P. contorta* and *P. trichocarpa* showed the lowest relative standard errors (RSE = 0.27 and 0.06, respectively) and smallest mean assignment errors (136 km and 15 km, respectively), consistent with their dense sampling and short distances between sampling locations. In contrast, *P. strobus* and *P. tremuloides* , characterized by sparser sampling and fewer individuals, exhibited higher RSE values (0.39 and 0.20, respectively). Interestingly, *P. mariana* achieved a low RSE (0.07) despite wider sampling gaps.

For *P. mariana* , KNN was the best‐performing model, achieving the lowest mean prediction error of 113 km when using the full set of markers. A sharp decline in error was observed up to approximately 5000 SNPs, beyond which additional markers did not substantially improve model accuracy (Figure 3). In *P. contorta* , LM, GB, and RF exhibited improved performance up to around 500 SNPs, after which GB consistently outperformed the other models. KNN, however, showed minimal improvement with additional markers and consistently produced poor predictions. Although LM displayed high variability in performance depending on marker count, it generally performed as well as GB when using the full dataset. This suggests that GB is the best algorithm in this case, as it achieves high accuracy with fewer markers. *P. strobus* exhibited trends similar to *P. contorta* , with relatively flat error curves, indicating that adding more markers did not significantly enhance prediction accuracy. Notably, this was the only species where LM outperformed all other algorithms. For *P. trichocarpa* , prediction error followed a multiplicative inverse relationship with the number of markers, particularly for KNN, stabilizing between 2000 and 5000 SNPs. The lowest overall prediction errors among all tested species were observed in *P. trichocarpa* , likely due to the high sampling density (mean distance between sampling locations = 260 km, Table 2), which captures the species' genetic diversity. In *P. tremuloides* , all models followed a general trend of decreasing error with additional markers, though high variability was evident, especially with smaller marker sets. No model consistently outperformed the others, except for KNN, which exhibited lower prediction errors.

Mean distance error differed between latitude and longitude predictions and across species (Figure S10). In general, mean prediction error was lower for latitude than for longitude, suggesting that geographic coordinates were more accurately predicted along the north–south axis than along the east–west axis. This pattern was particularly evident in *P. mariana* , where latitude predictions had moderate error and limited variability, whereas longitude predictions showed both higher error and greater variability. Similarly, *P. tremuloides* exhibited a larger discrepancy between latitude and longitude errors, with longitude predictions being especially inaccurate. In contrast, *P. trichocarpa* showed consistently low errors for both latitude and longitude, suggesting a well‐structured spatial genetic pattern in both dimensions.

## 4 Discussion

Traceability of wood and wood products is essential to promote sustainable forest management, combat illegal logging, and mitigate forest degradation (Godbout et al. 2018; Wiedenhoeft et al. 2019; Reboredo 2013). In this study, we leveraged datasets in which samples were obtained from both provenance trials and natural population collections, where the exact origin of each sample is known. We applied a bioinformatic pipeline incorporating various statistical algorithms to evaluate geographic assignment accuracy. Our results demonstrate that precise geographic assignment is achievable using genomic markers, with prediction errors as low as 15 km in the case of *Populus trichocarpa* (Figure 2). Such level of resolution is often lacking in studies based on chemical analyses, which primarily focus on taxonomic identification rather than geographic origin (Kitin et al. 2021; Bisht et al. 2022).

### 4.1 Factors Affecting Assignment Accuracy

When predicting a sample's origin, one can use either classification algorithms to assign it to predefined groups (discrete assignment) or regression‐based algorithms to estimate continuous variables, such as geographic coordinates (continuous assignment). Our study demonstrates that the success of geographic origin prediction, whether to a group or to specific coordinates, is strongly influenced by the species' evolutionary history. Preliminary results for lodgepole pine show higher assignment accuracy to genetic clusters compared to geographic clusters, a pattern consistent with the post‐glacial history of North American conifer species (Gamache et al. 2003; Lindbladh et al. 2003). We also observed a decline in assignment accuracy as the number of predefined groups increased, particularly when using geographically defined sampling location. This reduction likely reflects the fact that arbitrarily delineated groups do not necessarily correspond to underlying genetic structure, combined with ongoing gene flow among sampling locations and insufficient geographic separation for strong differentiation to emerge. Because individuals rarely belong unambiguously to discrete groups, group‐based assignment (genetic or geographic) can be limited. These limitations motivated our choice to instead predict continuous geographic coordinates, an approach that which better captures gradual genetic patterns and yields higher and more consistent predictive performance.

While most traceability studies rely on prior knowledge of the ecology and distribution of the species (Schroeder et al. 2016; Cronn et al. 2021), our bioinformatic pipeline for predicting geographic coordinates adopts a fully data‐driven approach. Specifically, it uses only genotype data and geographic sampling coordinates, without incorporating prior information on population genetic structure, evolutionary history, or environmental variables. In this context, any variation in assignment performance among species directly reflects the extent to which these implicit biological and historical factors are captured by the genetic data alone. *Picea mariana* exemplifies how evolutionary history can shape genetic structure and, in turn, influence assignment accuracy. Populations of this species exhibit a gradient of genetic differentiation resulting from historical processes such as glacial refugia and are further influenced by isolation by distance and local adaptation (Gamache et al. 2003). These processes have resulted in the formation of three major genetic groups that reflect postglacial recolonisation history across the species' range (Gérardi et al. 2010; Prunier et al. 2012). Similar patterns may be expected in other species with widespread continental distributions and pronounced isolation‐by‐distance effects.

In addition to evolutionary history, the source and sampling context of genetic material can influence geographic assignment performance across species. For some species, individuals originated from provenance trials, whereas others were sampled directly from natural stands. Although provenance trials can introduce potential sampling biases (Park and Rodgers 2023), such as the nonrandom selection of parent trees, the seed lots used to establish these trials are typically derived from open‐pollinated cones collected from multiple mother trees. Given the extensive pollen dispersal characteristic of many forest tree species, this approach generally promotes genetic mixing within regions, such that sampled genotypes remain broadly representative of regional genetic diversity, even if fine‐scale spatial structure may be partially reduced.

Both provenance trials and natural stand collections may also reflect accessibility bias, with sampling concentrated near roads or established sites. This can limit coverage of environmental extremes and reduce representation of spatial genetic heterogeneity across climatic or edaphic gradients sites (Aspalter et al. 2025). Consequently, assignment accuracy depends not only on the strength of underlying spatial genetic structure, but also on how well the sampling design captures the geographic distribution of allele frequencies across the species' range.

Taken together, these factors suggest that assignment accuracy reflects a combination of species‐specific evolutionary history, sampling design, and the degree to which environmental heterogeneity is represented in both the genetic data and spatial sampling framework. This integrative perspective is particularly important for data‐driven traceability approaches that do not rely on a priori biological information, as their performance ultimately depends on how well the genetic signal captures the spatial and adaptive structure present in natural populations.

We showed that increasing the number of genetic markers generally improves the accuracy of geographic predictions for unknown samples, but using as few as 2000 SNPs can already achieve quite precise assignments without any prior knowledge. A recent study using stable isotope ratio and trace element analysis in *Betula, Fagus, Pinus*, and *Quercus* reported prediction accuracies between 180 and 230 km for harvest location in Europe (Mortier et al. 2024). Another study applying machine learning to beech and oak genomic data achieved a precision of 55 and 263 km, respectively (Degen et al. 2025). In comparison, our models reached accuracies ranging between 15 km in *Populus trichocarpa* and 383 km in *P. tremuloides* . These results highlight the potential of genomic approaches to match and in some cases exceed isotope‐based methods for geographic assignment in trees.

Hybridization is common among closely related tree species, such as lodgepole pine and jack pine, particularly in regions where their ranges overlap (Cullingham et al. 2013), which could explain the low *F*_IS_ value observed for lodgepole pine. These hybrids possess genetic material from both parental species and are often difficult to distinguish using traditional methods like wood anatomy or chemical analysis. However, genetic markers provide a reliable means of hybrid identification (e.g., Talbot et al. 2012; Meirmans et al. 2014). Using lodgepole pine as a case study, we demonstrate that removing hybrid individuals can improve prediction accuracy, but only to a certain extent. Excessive removal reduces the available genetic diversity and geographic signal, ultimately diminishing model performance. This is likely because hybrids tend to occur in specific regions within the parental species' overlap, providing valuable geographic information that refines coordinate‐based predictions (Burns et al. 2019). Hybridization is not limited to temperate species; it also occurs in tropical trees, such as Indian teak ( *Tectona grandis* ) populations, underscoring the need to account for hybridization when predicting the geographic origin of imported tropical species (Balakrishnan et al. 2024).

### 4.2 Algorithm Performance

The algorithms producing the smallest mean distance between original and predicted coordinates were KNN and GB. KNN was the best performing algorithm in three out of five species. It is a nonparametric method, meaning it does not assume a specific functional form for the relationship between SNP data and geographic location (Guo et al. 2003). This flexibility is advantageous for tree species with complex evolutionary histories, including local adaptation, hybridization, and migration events. Our results show that, as the number of SNPs increases, the performance gap between KNN and the other models widens. This suggests that KNN benefits more from additional SNPs, likely because more SNPs enhance the resolution of genetic similarity, allowing KNN to make finer‐scale spatial predictions. In different species of the white oak subgenus, KNN was shown to be 100% accurate in predicting geographic origin (assignment to 3 discrete groups: Europe, Asia and North America) and robust to sequencing errors (Tang et al. 2018). GB yielded the best performance for both *Pinus* species. This may be due to their relatively weaker population structure compared to *Picea mariana* . Both *Pinus contorta* and *P. strobus* exhibit similar patterns in geographic prediction, with GF outperforming other algorithms, and no significant reduction in prediction error with the addition of more SNPs markers. This result could be attributed to the fact that these two *Pinus* species show a higher degree of plasticity in juvenile traits such as the timing of budburst or budset and height, and limited signs of local adaptation, especially when compared with *Picea mariana* (Li et al. 1997; Sniderhan et al. 2018; Bisbing et al. 2021; Silvestro et al. 2023). The superior performance of GB may therefore be explained by its ability to capture complex, nonlinear interactions and subtle patterns in the data, which is particularly useful for modeling species that combine high plasticity with moderate local adaptation (Thessen 2016). However, GB may suffer from overfitting when too many SNPs are included, leading to diminishing returns in prediction accuracy (Natekin and Knoll 2013). While we explored a broad range of hyper‐parameter combinations, yielding models that required substantial computational resources, we cannot entirely rule out the possibility that further optimization using even more extensive hyper‐parameter grids could improve RF model performance.

According to the maps of predicted vs. original coordinates, models tend to predict coordinates toward the center of the species distribution, though the extent varies by algorithm. This effect can be attributed to several factors. Tree‐based models like GB and RF iteratively minimize overall error, leading to a smoothing effect where predictions cluster around areas with higher training sample density (Natekin and Knoll 2013). Additionally, models like KNN, which rely on the proximity of known data points, may also bias predictions toward well‐represented regions (Weiss and Provost 2001). On the other hand, if genetic structure reflects historical dispersal patterns, predictions may be pulled toward regions of high genetic diversity, such as glacial refugia, where multiple lineages coexisted and hybridized (Shafer et al. 2010). This hypothesis suggests that the centralization effect may not only stem from algorithmic bias but also from biological and historical factors shaping population distributions.

Assignment is influenced by the evolutionary history and biology of the species under study (e.g., migration, dispersion) as well as by the sampling density. Although we have similar amounts of data (in terms of both SNPs and individuals), the geographic range covered by *Populus tremuloides* is much broader. The mean distance between sampling locations is approximately 1880 km for *P. tremuloides* , compared to just 260 km for *P. trichocarpa* , resulting in more spatially dispersed data for the former. In addition, *P. tremuloides* exhibits higher genetic diversity than *P. trichocarpa* as well as a different demographic history shaped during the Quaternary Ice Age (Wang et al. 2016). As expected, this contributes to larger prediction errors in the models, highlighting that accurate origin prediction for species with very wide distribution ranges, requires substantial sampling efforts (see next section).

### 4.3 Predictions Differ From Species to Species

Local adaptation is driven by different environmental factors in our five species: for example, *Populus trichocarpa* distribution ranges across several mountain chains (Geraldes et al. 2014), whereas *Picea mariana* might be less affected by elevation because it is distributed mostly north of the Rockies (Prunier et al. 2012). North America also spans across an aridity gradient with different tree genetic groups behaving differently in terms of local adaptation (Piper and Fajardo 2024). We found that *P. trichocarpa* and *P. mariana* had the smallest error indices, indicating that models were highly effective at predicting geographic origins in these species. The high predictive accuracy for *P. trichocarpa* is unsurprising given its high sampling density. In contrast, the strong performance for *P. mariana* is noteworthy, as this species has a wide distribution range with large geographic distances between sampling locations. This result suggests that despite extensive spatial separation, strong local adaptation in *P. mariana* enhances model performance by maintaining genetic structure correlated with geography (Lindbladh et al. 2003). For *Populus tremuloides* , the relatively low accuracy in predicting geographic coordinates compared to the other studied species could be attributed to its small sample size relative to its vast distribution range, which covers the majority of North America. Although the presence of four major genetic clusters likely facilitates assignment to broad geographic regions, limited genetic differentiation among populations within these clusters contrains the prediction of precise latitude and longitude. The exclusion of triploid genotypes likely increased assignment accuracy, as most genomic assignment methods assume diploidy and triploid individuals can bias allele‐frequency estimates. In addition, the inclusion of highly genetically distinct populations, such as the Lake Tahoe population, likely contributed to prediction accuracy. Overall, the lower assignment accuracy observed in *P. tremuloides* likely reflects the combination of extensive gene flow, high dispersal capacity and weak genetic differentiation among northern populations (Goessen et al. 2022). Higher prediction accuracy might be expected in regions such as the western United States, where population structure is more pronounced.

### 4.4 Key Recommendations and Perspectives

**FIGURE 4:**
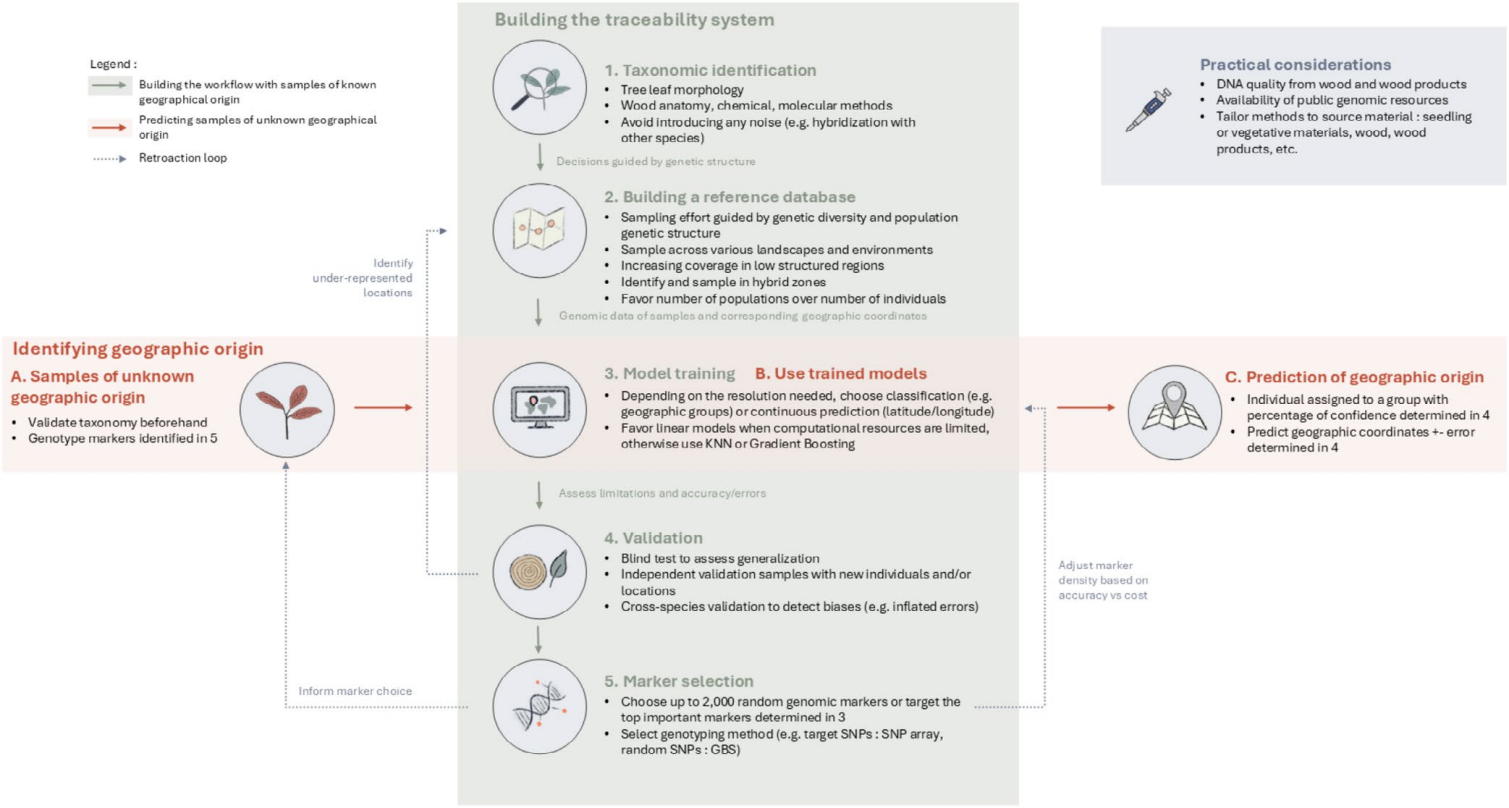
Practical guidelines for genomic‐based geographic assignment. This figure presents an iterative, decision‐driven workflow for *de novo* geographic assignment based on genomic data. Steps 1 to 5 describe the development of a traceability system, including taxonomic validation, reference database design guided by evolutionary and population genetic structure, model training tailored to prediction objectives and computational constraints, validation to assess generalization and potential biases, and marker selection to balance performance and cost. Once established, the selected marker set and trained models are applied to samples of unknown origin (steps A to C) to estimate geographic location and associated uncertainty. Feedback loops illustrate how validation results inform reference database refinement and optimisation of marker density. Although specific implementation may vary across biological systems, this framework provides a standardized foundation to improve assignment accuracy, robustness, and reproducibility. Beyond timber traceability, identifying the origin of native trees and seedlings is critical for climate adaptation. Programs like Canada's 1 Billion Trees (https://www.canada.ca/en/campaign/2‐billion‐trees/2‐billion‐trees‐program.html) support afforestation to enhance carbon sequestration (Domke et al. 2020; Ontl et al. 2020). Assisted migration, whether via gene flow or colonization (Aitken et al. 2008; Aitken and Whitlock 2013; Duchesne et al. 2022) requires accurate knowledge of genetic origin to ensure climate suitability and long‐term survival (Williams and Dumroese 2013; Pedlar and McKenney 2017; O'Neill and Gómez‐Pineda 2021). In the long term, increasing gene flow from assisted migration might weaken population structure, complicating future assignment (Bohonak 1999), seedlings can already be traced back to their parental origin in some seedling production systems, particularly when a traceability system is in place (e.g., Godbout et al. 2017) or when genomic selection is included prior material deployment (Lenz et al. 2020; Laverdière et al. 2022). Traceability also helps mitigate phytosanitary risks linked to the movement of wood and plant material, which can spread invasive pests and pathogens (Solano et al. 2021), and strong systems aligned with biosecurity regulations reduce this threat (Hamelin and Roe 2019).

Based on our study, we propose the following guidelines for optimizing genomic‐based geographic assignment (Figure 4). While these recommendations may not be universally applicable to all species, they provide a solid foundation for future assignment analyses and can help improve model performance and efficiency.

Determining whether wood originates from plantations, natural stands, or introduced species adds an additional layer of complexity to traceability efforts. Some protected species, such as mahogany (*Swietenia* spp.), may be legally cultivated in plantations (Richardson et al. 2024), while introduced species like Norway spruce ( *Picea abies* ), though widely planted in North America, may be excluded from certain markets due to regulatory criteria (National Lumber Grades Authority (NLGA) 2019, 2022, https://nlga.org/en/). A complementary approach combining genetic tools and geographic provenance information can help better distinguish sources of supply. A proactive approach could involve assigning barcodes to reforested species (provenance, families, etc.) from the outset, based on well‐documented genetic and geographic origin. This strategy would help anticipate regulatory requirements and support the integration of these species into certified supply chains.

The precision required for wood traceability depends on the objective (Godbout et al. 2018). Customs enforcement may only need species or country‐level identification, while forest certification and sustainability efforts demand finer‐scale geographic precision. Though predicting exact coordinates requires more data and sophisticated methods, appropriate tools can achieve all these goals. This approach relies on species‐specific spatial or genetic structure and large datasets (Godbout et al. 2018; Emmert‐Streib et al. 2020), but growing public genomic databases and falling sequencing costs make implementation increasingly viable (Kodama et al. 2012; Peng et al. 2023). While degraded DNA in wood poses challenges, robust SNP genotyping platforms make genomic identification feasible, as shown in *Acer macrophyllum* (Dormontt et al. 2020). Nevertheless, genomics traceability will not be universally suitable; methods must match traceability goals and species biology (Manel et al. 2005). Machine learning models also incur computational and environmental costs (Grealey et al. 2022), to mitigate this, we provide guidelines (Figure 4) and ranked SNP lists for five focal species.

## Funding

Ilga Porth and Nathalie Isabel acknowledge funding support through the Génome Québec Genomics Integration Program grant “Genomics‐Informed Forest Certification and Wood Traceability Tools and Protocols” 2022–2025. Nathalie Isabel further acknowledges funding support from the Genomics Research and Development Initiative (GRDI). Ilga Porth also acknowledges funding support for the original genotype data generation for *Populus* spp. through an NSERC Discovery grant (*RGPIN/04748‐2017*). Support from Digital Alliance Canada for providing computational resources is also acknowledged.

## Conflicts of Interest

The authors declare no conflicts of interest.

## Supporting information


**Figure S1:** Geographic distribution of genetic structure in *Pinus contorta* . Each pie chart represents one of the 281 sampled lodgepole pine populations, positioned according to its geographic coordinates. The colors within each pie chart correspond to the proportion of individuals assigned to each of the three genetic clusters for A, *K* = 2 and B, *K* = 4 as inferred by STRUCTURE analysis.
**Figure S2:** Geographic clustering (*n* = 4) of lodgepole pine populations based on spatial coordinates. Each point represents one of the 281 sampled populations and is positioned according to its geographic location. Colors indicate membership to one of four spatial clusters identified using KNN clustering based on latitude and longitude.
**Figure S3:** Effect of hybrid filtering on random forest assignment accuracy across varying numbers of SNPs. Individuals were grouped into genetic clusters using Structure for *K* = 2, 3, and 4. For each *K*, individuals with less than 80% membership to any one cluster were considered hybrids and excluded. RF models were then trained and tested using only the remaining individuals. The *y*‐axis shows the proportion of individuals correctly assigned to their population of origin (accuracy), and the *x*‐axis indicates the number of SNPs used in model training. Each line represents a different value of *K*.
**Figure S4:** Distribution of spatial prediction errors for the best‐performing model per species. Boxplots show the distribution of mean geographic error distances (in km) for individuals of our five tree species: *Populus trichocarpa* , *Picea mariana* , *Pinus strobus* , *Populus tremuloides* , and *Pinus contorta* .
**Figure S5:** Geographic assignment of *Picea mariana* individuals using genomic prediction. Observed sampling locations (circles) are connected by lines to their respective predicted geographic coordinates, inferred using the best‐performing machine learning model trained on genomic data. The spatial displacement between observed and predicted points illustrates the model's accuracy in capturing spatial genetic structure across the study region.
**Figure S6:** Geographic assignment of *Pinus contorta* individuals using genomic prediction. Observed sampling locations (circles) are connected by lines to their respective predicted geographic coordinates, inferred using the best‐performing machine learning model trained on genomic data. The spatial displacement between observed and predicted points illustrates the model's accuracy in capturing spatial genetic structure across the study region.
**Figure S7:** Geographic assignment of *Pinus strobus* individuals using genomic prediction. Observed sampling locations (circles) are connected by lines to their respective predicted geographic coordinates, inferred using the best‐performing machine learning model trained on genomic data. The spatial displacement between observed and predicted points illustrates the model's accuracy in capturing spatial genetic structure across the study region.
**Figure S8:** Geographic assignment of *Populus tremuloides* individuals using genomic prediction. Observed sampling locations (circles) are connected by lines to their respective predicted geographic coordinates, inferred using the best‐performing machine learning model trained on genomic data. The spatial displacement between observed and predicted points illustrates the model's accuracy in capturing spatial genetic structure across the study region.
**Figure S9:** Geographic assignment of *Populus trichocarpa* individuals using genomic prediction. Observed sampling locations (circles) are connected by lines to their respective predicted geographic coordinates, inferred using the best‐performing machine learning model trained on genomic data. The spatial displacement between observed and predicted points illustrates the model's accuracy in capturing spatial genetic structure across the study region.
**Figure S10:** Comparison of geographic prediction errors for latitude and longitude across species. The left panel shows the distribution of prediction errors (in km) for latitude, and the right panel shows the same for longitude. Each boxplot represents the error distribution for one species, based on predictions from the best‐performing model for that species.
**Table S1:** Algorithms and ranges of hyperparameters tested to optimize the machine‐learning models predicting latitude and longitude independently. The ranges of hyperparameters were searched using a grid search approach with a 5‐fold cross‐validation implemented in the *GridSearchCV* python function (scikit‐learn library v.1.1.2).
**Table S2:** Algorithms and hyperparameters for models achieving the highest performances within each species datasets.

## Data Availability

Lists of SNPs ordered by importance in best models for all species and code are available on github https://github.com/jprunier‐ulaval/ForestTreesML.
