# Supplementary material for: Harnessing Genomic Information for Identifying the Geographic Origin of Five North American Tree Species in Trade
Source: Evol Appl. 2026 Jul 30;19(8):e70295. doi: 10.1111/eva.70295 (PMC13420222; doi:10.1111/eva.70295)
Supplement: Supplementary file 1 — Figure S1: Geographic distribution of genetic structure in Pinus contorta . Each pie chart represents one of the 281 sampled lodgepole pine populations, positioned according to its geographic coordinates. The colors within each pie chart correspond to the proportion of individuals assigned to each of the three genetic clusters for A, K = 2 and B, K = 4 as inferred by STRUCTURE analysis. Figure S2: Geographic clustering (n = 4) of lodgepole pine populations based on spatial coordinates. Each point represents one of the 281 sampled populations and is positioned according to its geographic location. Colors indicate membership to one of four spatial clusters identified using KNN clustering based on latitude and longitude. Figure S3: Effect of hybrid filtering on random forest assignment accuracy across varying numbers of SNPs. Individuals were grouped into genetic clusters using Structure for K = 2, 3, and 4. For each K, individuals with less than 80% membership to any one cluster were considered hybrids and excluded. RF models were then trained and tested using only the remaining individuals. The y‐axis shows the proportion of individuals correctly assigned to their population of origin (accuracy), and the x‐axis indicates the number of SNPs used in model training. Each line represents a different value of K. Figure S4: Distribution of spatial prediction errors for the best‐performing model per species. Boxplots show the distribution of mean geographic error distances (in km) for individuals of our five tree species: Populus trichocarpa , Picea mariana , Pinus strobus , Populus tremuloides , and Pinus contorta . Figure S5: Geographic assignment of Picea mariana individuals using genomic prediction. Observed sampling locations (circles) are connected by lines to their respective predicted geographic coordinates, inferred using the best‐performing machine learning model trained on genomic data. The spatial displacement between observed and predicted points illustrates th [file EVA-19-e70295-s001.docx]

Supplementary material

Supplementary figures
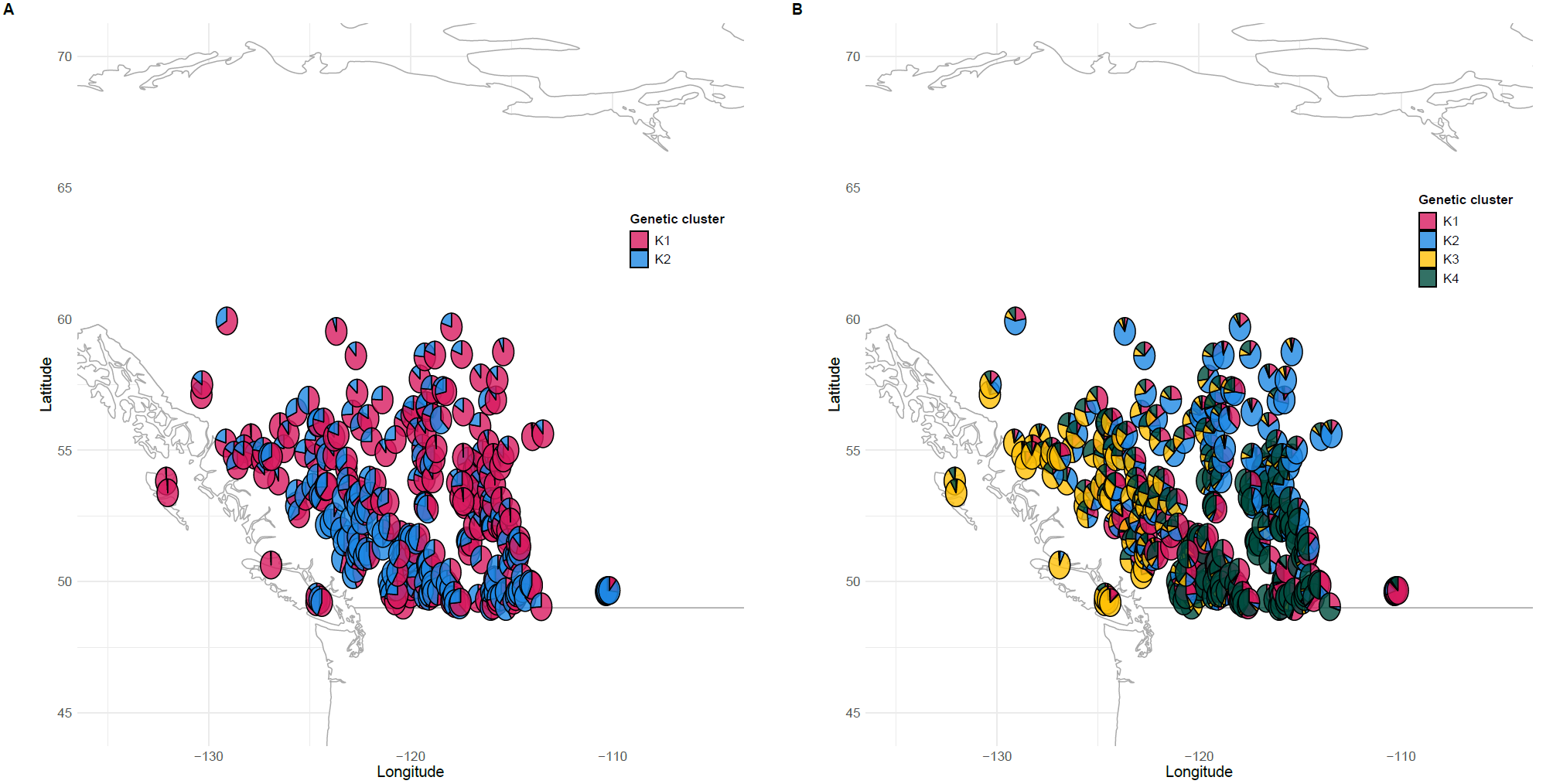


**Sup. Fig. 1**. **Geographic distribution of genetic structure in *P. contorta***. Each pie chart corresponds to one of the 281 sampled lodgepole pine populations, positioned by geographic coordinates. The colors within each pie chart represent the proportion of individuals assigned to each of the inferred genetic clusters STRUCTURE analysis (**A**, K = 2; **B**, K = 4)


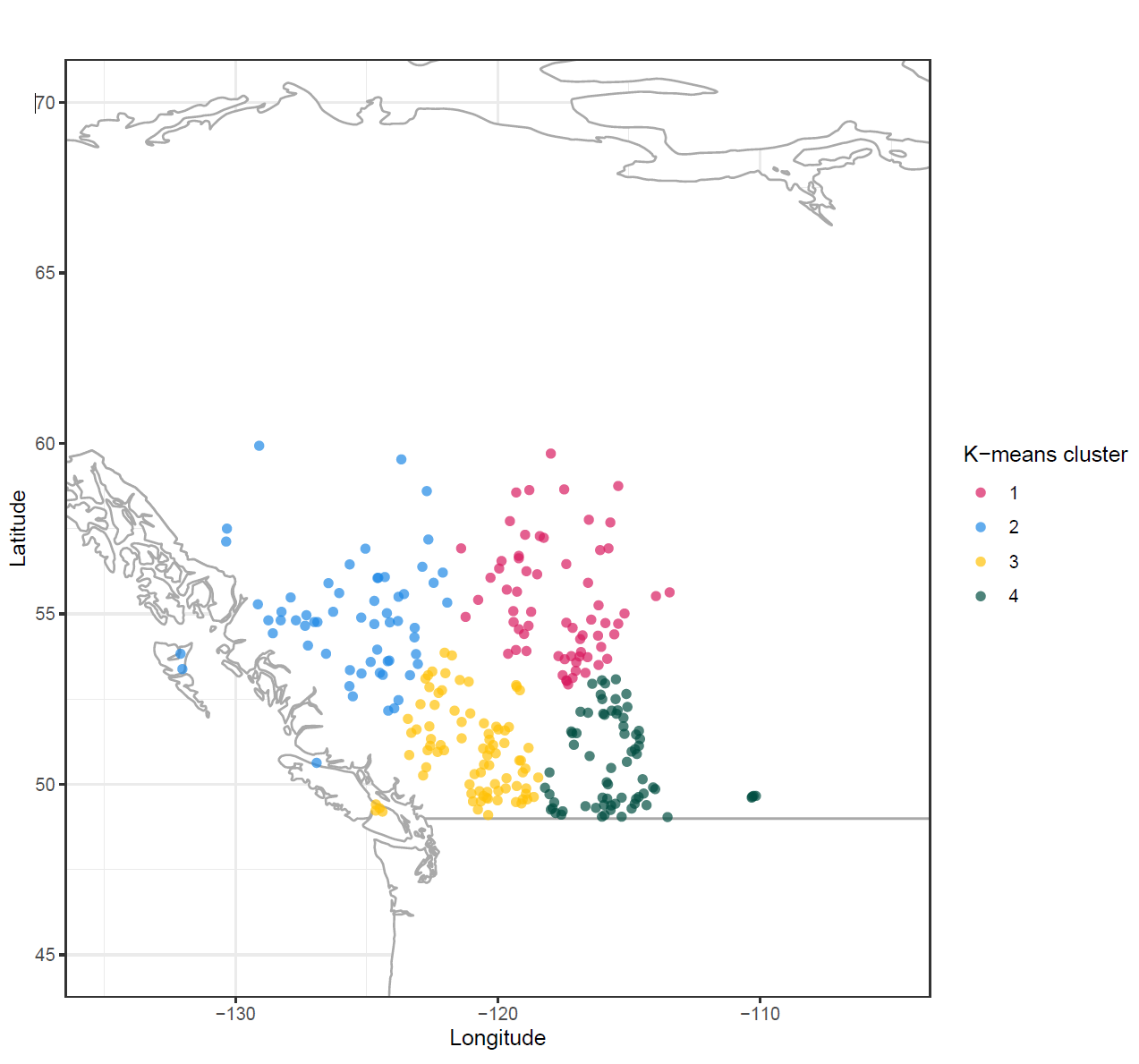


**Sup. Fig. 2**. **Geographic clustering (n = 4) of lodgepole pine populations based on spatial coordinates.** Each point corresponds to one of the 281 sampled populations, positioned by latitude and longitude with colors indicating membership to one of four spatial clusters identified using KNN clustering.


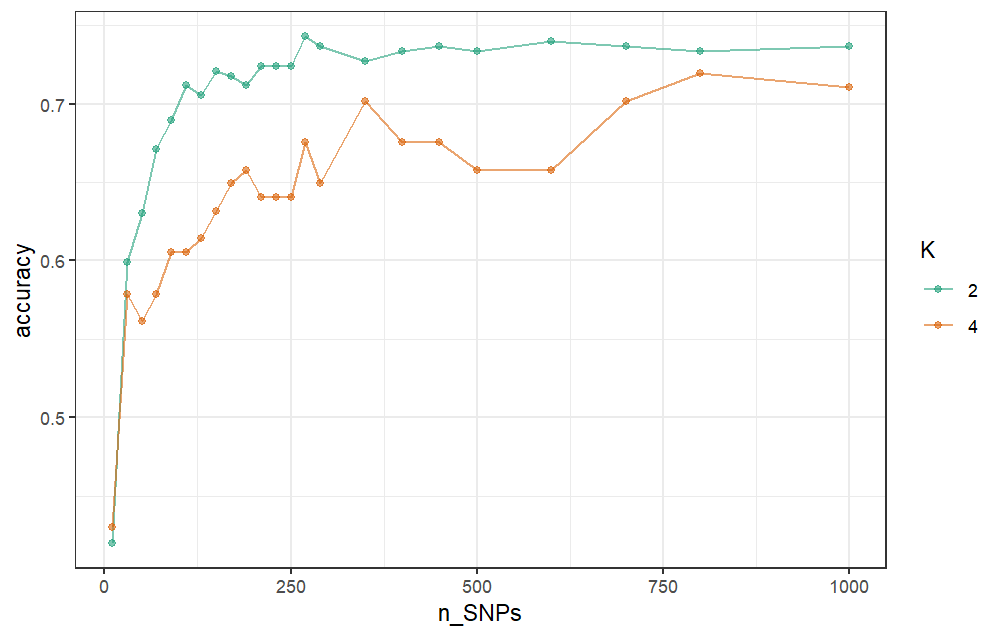


**Sup. Fig. 3**. **Effect of hybrid filtering on random forest (RF) assignment accuracy across varying numbers of SNPs.** Individuals were grouped into genetic clusters using STRUCTURE for K = 2, 3, and 4. For each K, individuals with less than 80% ancestry membership in any single cluster were classified as hybrids and excluded. RF models were then trained and tested using only the remaining individuals. The y-axis shows the proportion of individuals correctly assigned to their population of origin (accuracy), and the x-axis indicates the number of SNPs used in model training. Each line represents a different value of K.


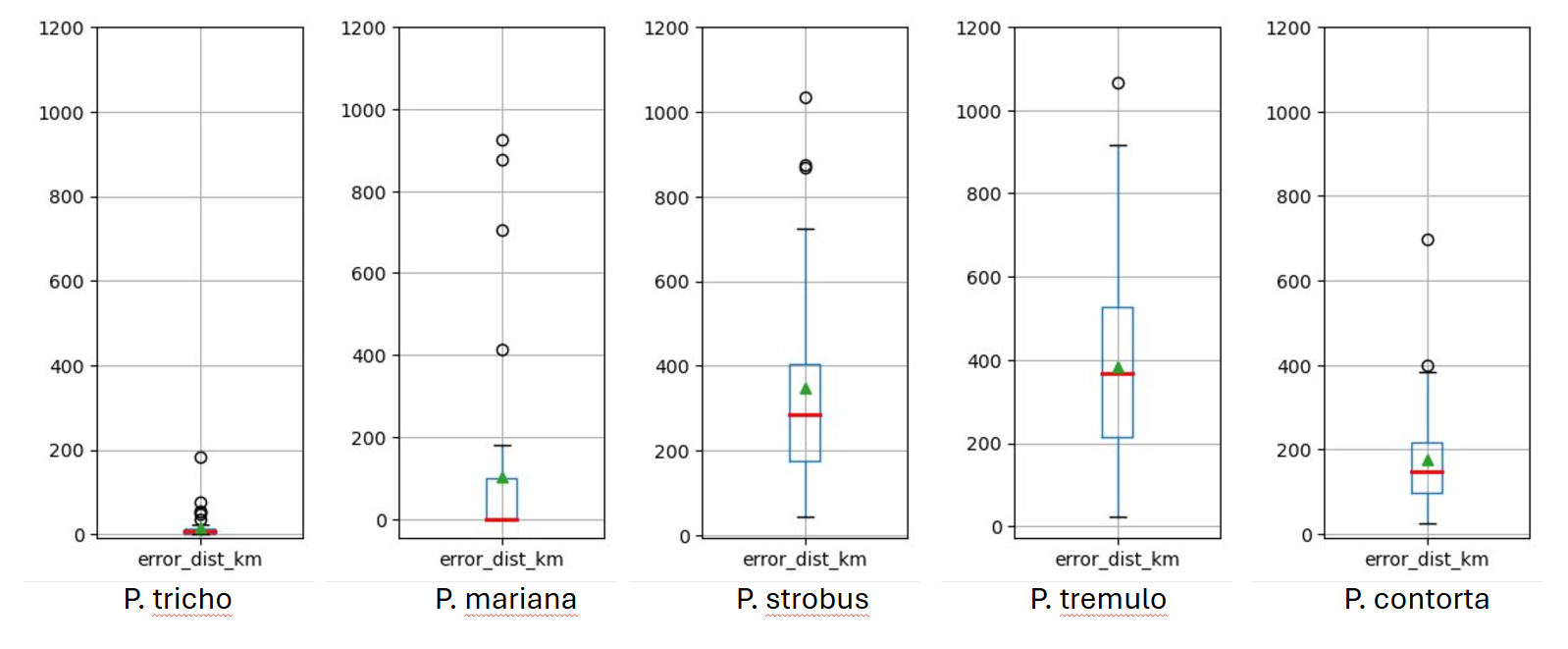
**Sup Fig 4. Distribution of spatial prediction errors from the best-performing model for each species.** Boxplots depict mean geographic error distances (in km) for individuals from five tree species: *P. trichocarpa*, *P. mariana*, *P. strobus*, *P. tremuloides*, and *P. contorta*.


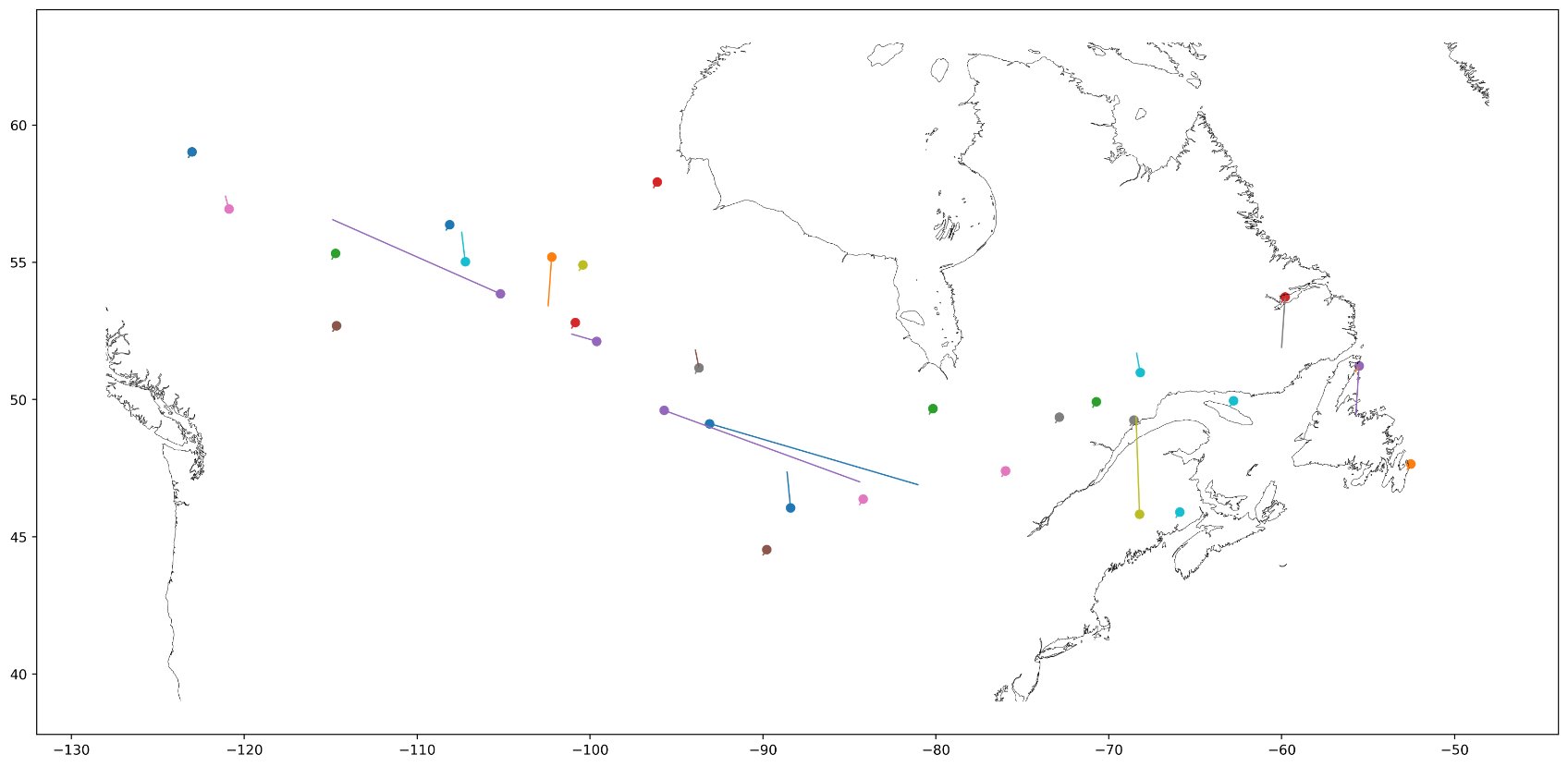


**Sup. Fig. 5 . Geographic assignment of *P. mariana* individuals using genomic prediction.** Observed sampling locations (circles) are connected to their corresponding predicted geographic coordinates by lines, inferred using the best-performing machine learning model trained on genomic data. The spatial displacement between observed and predicted locations illustrates the model’s accuracy in capturing spatial genetic structure across the study region.


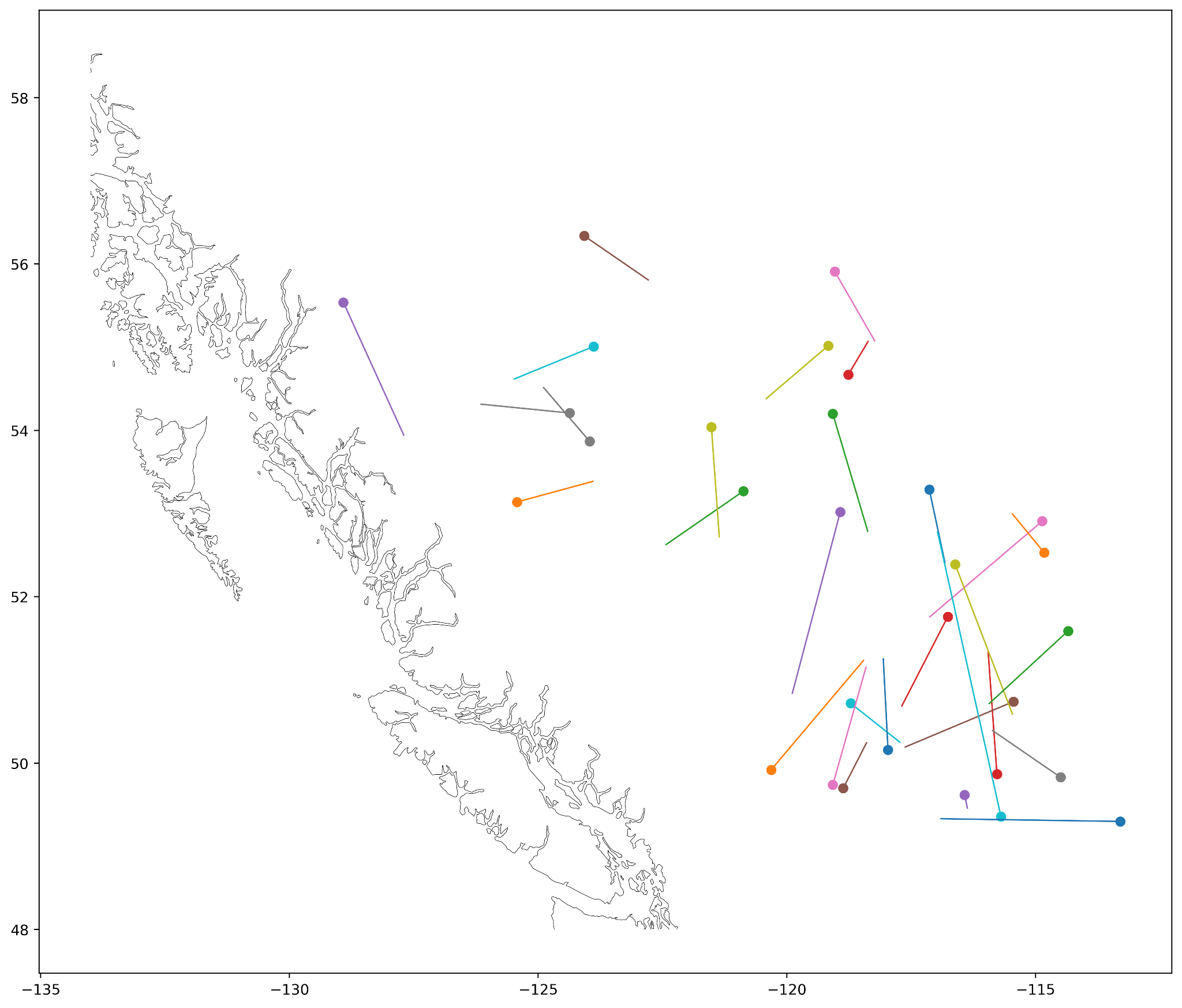


**Sup Fig 6. Geographic assignment of *P. contorta* individuals using genomic prediction.** Observed sampling locations (circles) are connected to their corresponding predicted geographic coordinates by lines, inferred using the best-performing machine learning model trained on genomic data. The spatial displacement between observed and predicted locations illustrates the model’s accuracy in capturing spatial genetic structure across the study region.


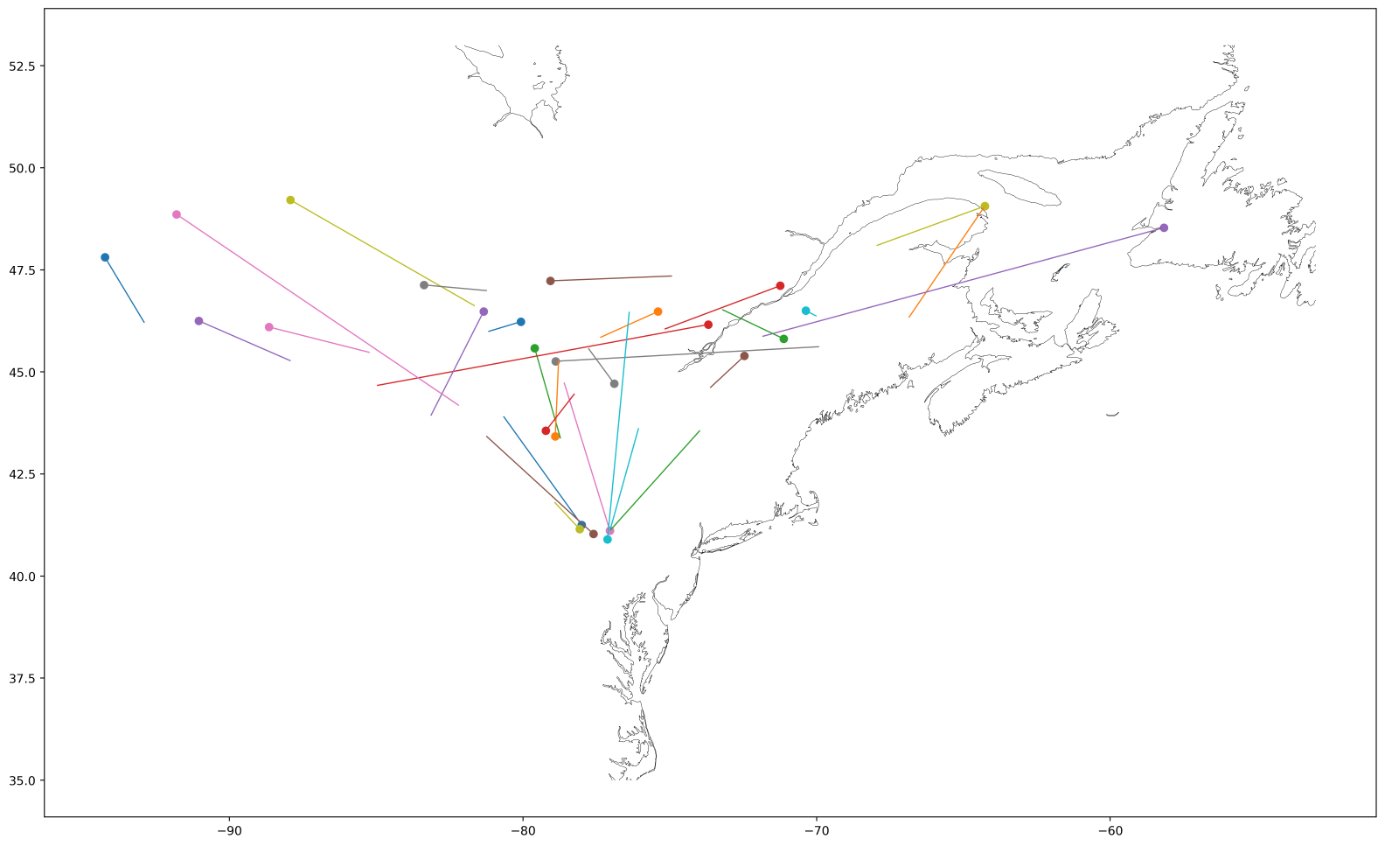


**Sup Fig 7. Geographic assignment of *P. strobus* individuals using genomic prediction.** Observed sampling locations (circles) are connected to their corresponding predicted geographic coordinates by lines, inferred using the best-performing machine learning model trained on genomic data. The spatial displacement between observed and predicted locations illustrates the model’s accuracy in capturing spatial genetic structure across the study region.


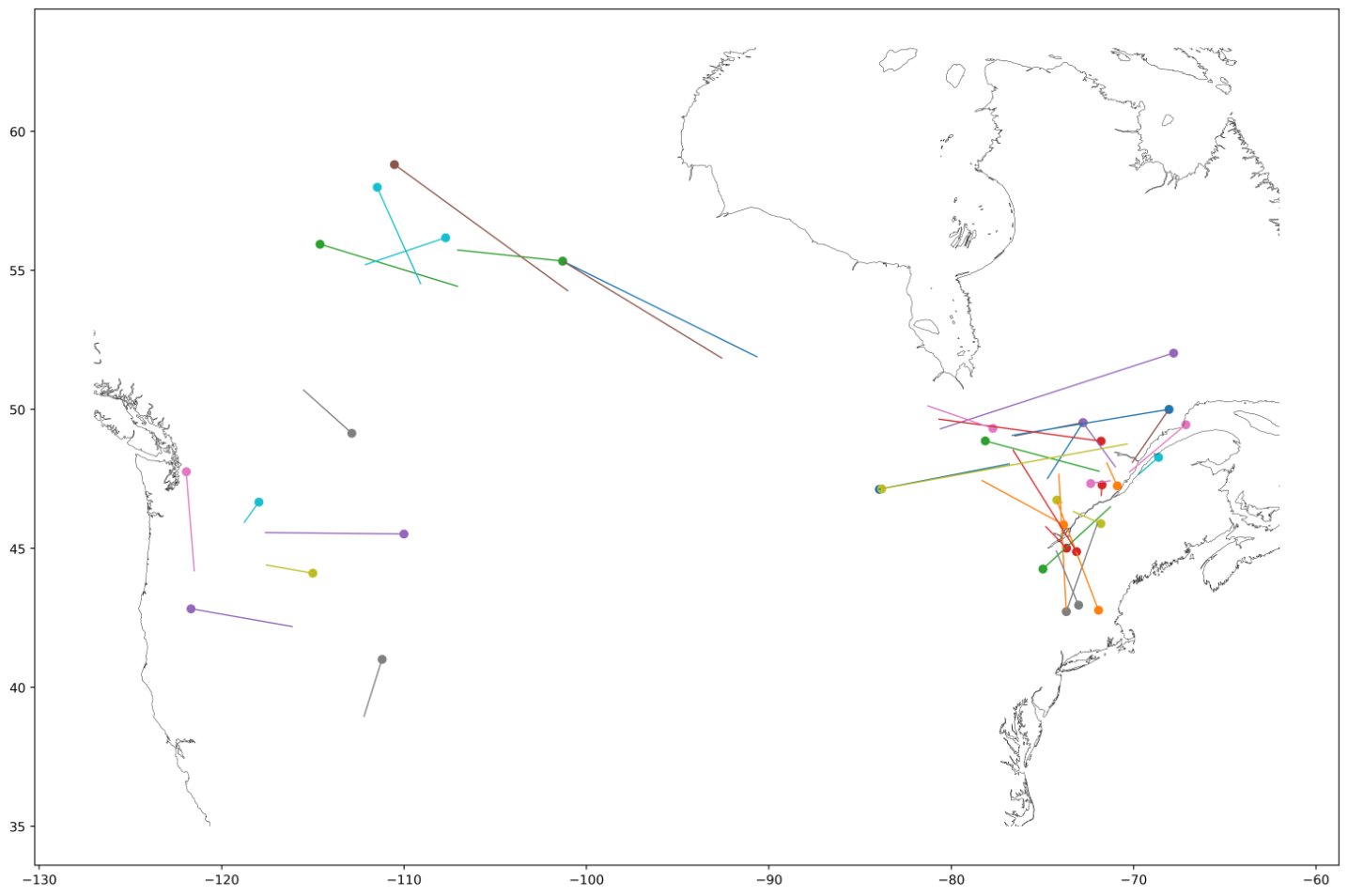


**Sup Fig 8. Geographic assignment of *P. tremuloides* individuals using genomic prediction.** Observed sampling locations (circles) are connected to their corresponding predicted geographic coordinates by lines, inferred using the best-performing machine learning model trained on genomic data. The spatial displacement between observed and predicted locations illustrates the model’s accuracy in capturing spatial genetic structure across the study region.


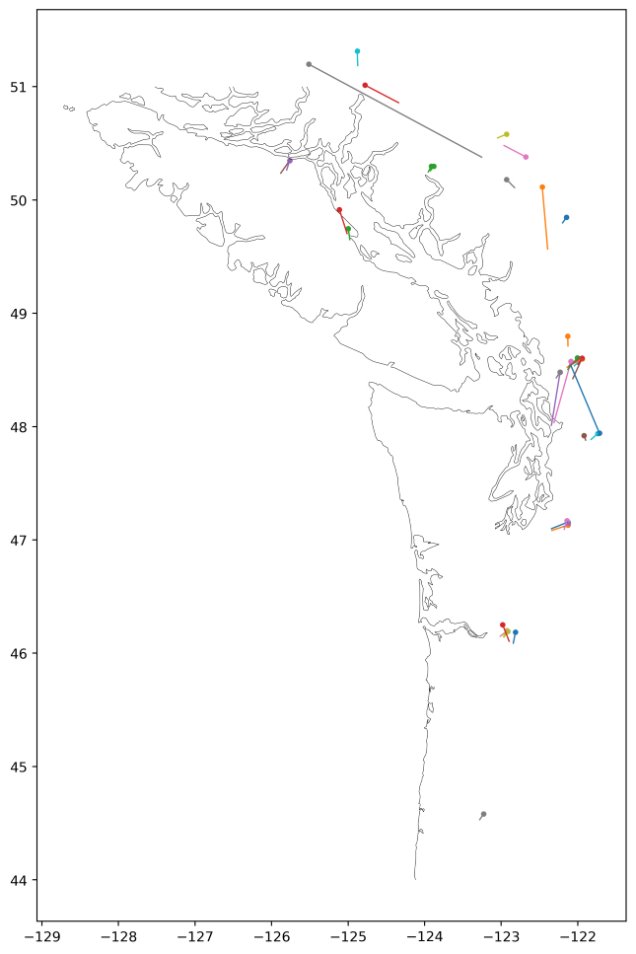


**Sup. Fig. 9. Geographic assignment of *P. trichocarpa* individuals using genomic prediction.** Observed sampling locations (circles) are connected to their corresponding predicted geographic coordinates by lines, inferred using the best-performing machine learning model trained on genomic data. The spatial displacement between observed and predicted locations illustrates the model’s accuracy in capturing spatial genetic structure across the study region.


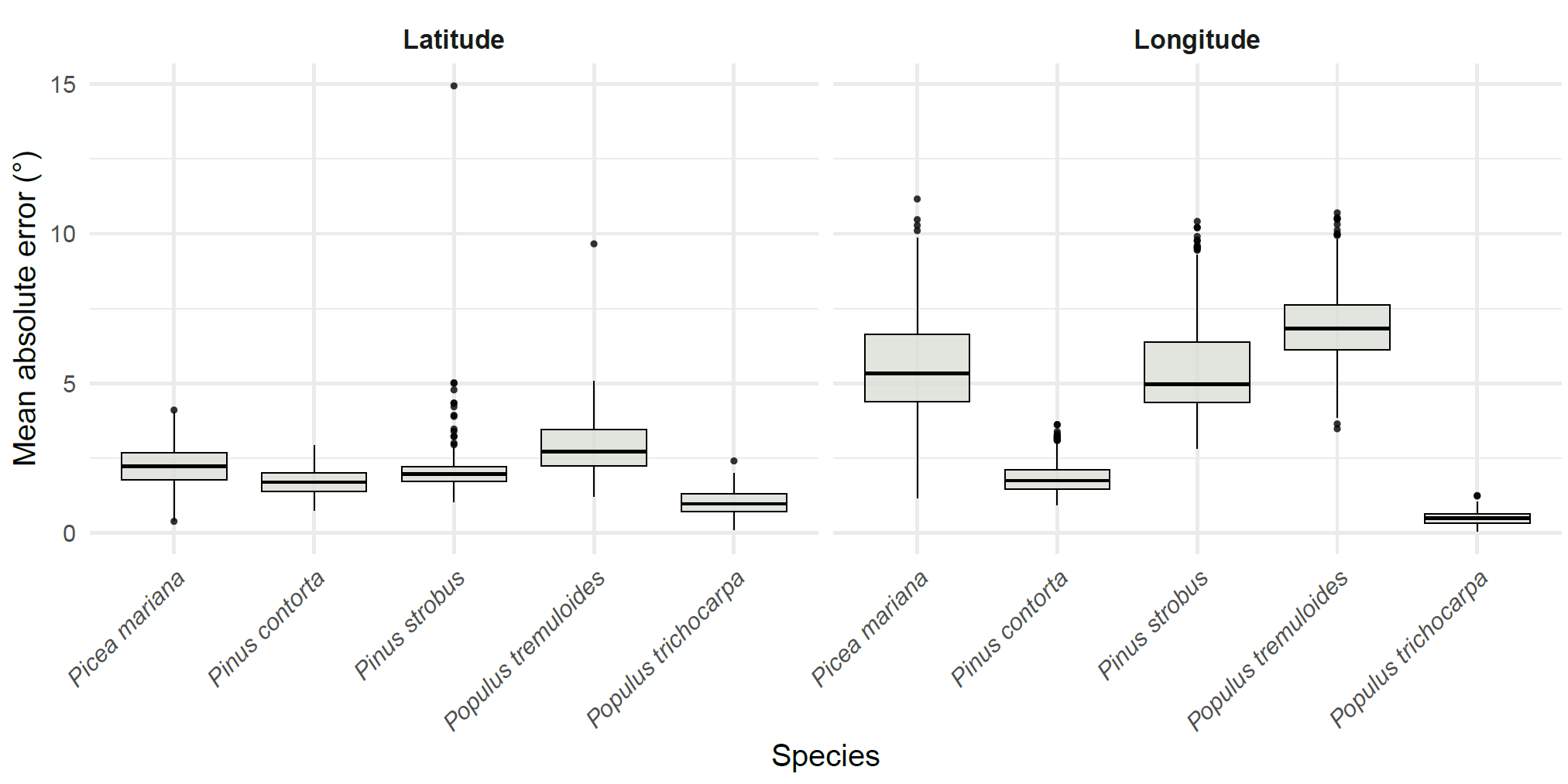


**Sup. Fig 10. Comparison of geographic prediction errors for latitude and longitude across species**. The left panel shows the distribution of latitude prediction errors (in km), while the right panel shows corresponding distribution for longitude. Each boxplot represents the error distribution for a single species, based on predictions from the best-performing model for that species.

**Sup. Table 1. Algorithms and ranges of hyperparameters tested to optimize machine-learning models predicting latitude and longitude independently.** Hyperparameter ranges were explored using a grid search approach with 5-fold cross-validation implemented via the *GridSearchCV* function in Python (scikit-learn library v.1.1.2)

| Algorithm | Hyper-parameter ranges |
| --- | --- |
| Linear | *none* |
| Random-Forest | 'min_samples_leaf':[1,2,5]  'n_estimators':[1000,2000,5000]  'min_samples_split':[2,5,10] |
| Gradient-Boosting | 'learning_rate':[0.01,0.1,1,10,100]  'n_estimators':[10,50,100,200,400,500,1000] |
| K-Nearest-Neighbours | 'n_neighbors':[1,3,5,10,15]  'weights':['uniform','distance']  'algorithm':['auto','ball_tree','kd_tree','brute'],  'leaf_size':[1,10,30,100],  'p':[1,2] |

**Sup. Table 2. Algorithms and hyperparameters associated with top-performing models for each species datasets**

| *Species* | Best algorithm | Hyper-parameters selected for the best- performing model predicting latitude | Hyper-parameters selected for the best performing model predicting longitude |
| --- | --- | --- | --- |
| *Picea mariana* | K-Nearest-Neighbours | 'algorithm' = 'auto'  'leaf_size'=1  'n_neighbors' = 3  'p' = 1  'weights' = ‘distance' | 'algorithm' = 'ball_tree'  'leaf_size' = 30  'n_neighbors' = 3  'p' = 1  'weights' = 'distance' |
| *Populus trichocarpa* | K-Nearest-Neighbours | 'algorithm' = 'auto'  'leaf_size' = 1  'n_neighbors' = 3  'p' = 1  'weights' = 'distance' | 'algorithm' = 'auto' 'leaf_size' = 1 'n_neighbors' = 1  'p' = 1  'weights' = 'uniform' |
| *Populus tremuloides* | K-Nearest-Neighbours | 'algorithm' = 'auto' 'leaf_size' = 1  'n_neighbors' = 15  'p' = 2  'weights' = 'distance' | 'algorithm' = 'ball_tree' 'leaf_size' = 1  'n_neighbors' = 10  'p' = 2  'weights' = 'distance' |
| *Pinus contorta* | Gradient-Boosting | 'learning_rate' = 0.1  'n_estimators' = 500 | 'learning_rate' = 0.1  'n_estimators' = 5000 |
| *Pinus strobus* | Gradient-Boosting | 'learning_rate' = 0.1  'n_estimators' = 400 | 'learning_rate' = 0.1  'n_estimators' = 2000 |

Supplementary Methods

Black spruce and eastern white pine dataset collection and sequencing

Between 2014 and 2019, we collected samples from 70 provenances of black spruce (*Picea mariana* (Mill.) B.S.P.) and 110 provenances of eastern white pine (*Pinus strobus* L.), totaling 2,483 specimens for black spruce and 617 specimens for eastern white pine, encompassing a large part of the species' entire natural range. Black spruce samples were gathered from common garden provenance tests located in Chibougamau (QC), Acadia (NB), Mont-Laurier (QC), and Peace River (AB), all of which were initially established in 1974 or 1975 as part of the Range-Wide Provenance Study, with seeds sown in 1970 (Morgenstern, 1978). Eastern white pine samples were collected from various sources in the Québec City area, including seed orchard located at Cap-Tourmente (47.06°N, 70.81°W; elevation 20 m), a provenance-progeny test established in Valcartier (46.98°N, 71.47°W; elevation 222 m), and seedlings grown from seeds obtained from the Canadian Forest Service’s National Tree Seed Centre (Atlantic Forestry Centre, Fredericton, New Brunswick, Canada) and sown in 2012 (Nadeau et al. 2015). Black spruce and eastern white pine samples were prepared as follows. The DNA required for sequencing was extracted from 30-50 mg of either frozen needle tissue or cambial tissue (for Acadia and Peace River sites) with a Nucleospin 96 Plant II kit (Macherey-Nagel, Bethlehem, PA) using the centrifugation processing protocol with a cell lysis step with PL2 buffer for 1 h at 65 °C, Cambium/phelloderm tissue was obtained from bark samples collected with a 1 cm diameter punch sterilized with 70% alcohol between each sample. Prior to extraction, all tissues were ground to powder with a Mixer Mill MM300 (Retsch GmbH, Haan, Germany) after being plunged into liquid nitrogen for two minutes (this process was repeated twice). Sequencing and Genotyping For single nucleotide polymorphisms (SNPs) discovery, we employed the DArTseq™ method, which shares similarities with genotyping-by-sequencing (GBS) but includes a complexity reduction step targeting low-copy sequences within the genome. Our procedure involved digesting genomic DNA with PstI and MseI restriction enzymes, ligating barcoded adapters, amplifying the resulting products via PCR, and sequencing them using a HiSeq 2500 system. Sequencing was conducted in one batch for *P. strobus* and three for *P. mariana*. This entire process was conducted by Diversity Arrays Technology, headquartered in Bruce, Australia, as described by Kilian et al. in 2012. Our SNP analysis focused exclusively on dinucleotide SNPs for subsequent investigation. The DNA plates containing extracted samples were sent and processed in three consecutive years: 2018, 2019, and 2020, referred to herein as batches 1, 2, and 3. Genotypes were generated separately for each of these three batches, and at the conclusion of the project, all sequences produced were merged and clustered together to create the final dataset for analysis.

Quaking aspen dataset collection and sequencing

Leaf samples were collected from 1,903 *Populus tremuloides* trees covering the entire natural distribution (Canada, U.S. and Mexico), and representing 110 natural populations, through a collaboration of various researchers (Goessen et al., 2022; Goessen et al., 2025, under review). DNA extraction was performed on dried leaf tissue using the Nucleospin 96 Plant II kit (Macherey-Nagel, Bethlehem, PA, U.S.), following the manufacturer’s protocol with modifications to the cell lysis step. Specifically, the PL2 buffer was heated at 65°C for 2 hours, rather than the recommended 30 minutes. Two separate libraries were prepared: one with samples at 10 ng/μL, and another with samples at both 10 ng/μL and 3 ng/μL concentrations. Illumina triple digest Genotyping-by-Sequencing (3D-GBS) libraries were prepared at the Plateforme d’Analyses Génomiques, Institut de Biologie Intégrative et des Systèmes (IBIS, Université Laval, Québec, Canada), using the protocol described by Poland et al. (2012), with modifications to optimize the number of SNPs. These modifications included: (i) the addition of the restriction enzyme NsiI to the original PstI/MspI combination, and (ii) the incorporation of a size selection step (150-400 bp) using a blue Pippin (SAGE Sciences) with elution times set between 50 and 65 minutes on a 2% agarose gel, prior to final library amplification. Barcodes were incorporated following the method outlined in Colston-Nepali et al. (2019). Sequencing was performed on an Illumina Novaseq6000 S4 (1 lane) at the Centre d’expertise et de services Génome Québec in Montréal, Canada, yielding paired-end 150-bp reads in FastQ format.

Data processing and SNP VCF file generation were carried out using STACKS version 2.4 (Catchen et al., 2013) and the stacks_workflow pipeline (<https://github.com/enormandeau/stacks_workflow>). Raw sequencing data was cleaned with Cutadapt version 2.7 (Martin, 2011), and samples were processed using the process_radtags function from STACKS. Hereafter, reads were aligned to the genome with bwa version 0.7.17 (Li & Durbin, 2010) and samtools version 1.8 (Li et al., 2009). SNPs were called with the STACKS2 pipeline using a novel *Populus tremuloides* reference genome (constructed using PacBio HiFi sequencing, Goessen et al. in preparation).

The resulting vcf datafile contained 1,666,993 SNPs and was filtered for triploid individuals (see Goessen et al., 2022). Hereafter we filtered the data using the pipeline available at <https://github.com/enormandeau/stacks_workflow>, which includes a relatedness filter of 0.3 to exclude clonal genotypes and a minor allele frequency > 0.033 (described in detail in Goessen et al., 2025 (under review)). For the analysis, the Mexican cluster was removed from the dataset because it is genetically distinct from the remaining genetic cluster of *P. tremuloides* and we rather wanted to focus on assignment within Canada /US.
